# Evolving AIDS- and non-AIDS Mortality and Predictors in the PISCIS Cohort of People Living With HIV in Catalonia and the Balearic Islands (Spain), 1998–2020

**DOI:** 10.1093/ofid/ofae132

**Published:** 2024-03-08

**Authors:** Daniel K Nomah, Suju Jamarkattel, Andreu Bruguera, Sergio Moreno-Fornés, Yesika Díaz, Lucía Alonso, Jordi Aceitón, Josep M Llibre, Pere Domingo, Maria Saumoy, Francesc Homar, Francisco Fanjul, Jordi Navarro, Lorena de la Mora, Hernando Knobel, Amat Orti, Raquel Martin-Iguacel, José M Miró, Jordi Casabona, Juliana Reyes-Urueña

**Affiliations:** Departament de Salut, Centre d’Estudis Epidemiològics sobre les Infeccions de Transmissió Sexual i Sida de Catalunya (CEEISCAT), Barcelona, Spain; Institut d'Investigació Germans Trias i Pujol (IGTP), Barcelona, Spain; Departament de Salut, Centre d’Estudis Epidemiològics sobre les Infeccions de Transmissió Sexual i Sida de Catalunya (CEEISCAT), Barcelona, Spain; Departament de Salut, Centre d’Estudis Epidemiològics sobre les Infeccions de Transmissió Sexual i Sida de Catalunya (CEEISCAT), Barcelona, Spain; Institut d'Investigació Germans Trias i Pujol (IGTP), Barcelona, Spain; CIBER Epidemiologia y Salud Pública (CIBERESP), Barcelona, Spain; Departament de Pediatria, d’Obstetrícia i Ginecologia i de Medicina Preventiva i de Salut Publica, Universitat Autònoma de Barcelona, Bellaterra, Spain; Departament de Salut, Centre d’Estudis Epidemiològics sobre les Infeccions de Transmissió Sexual i Sida de Catalunya (CEEISCAT), Barcelona, Spain; Institut d'Investigació Germans Trias i Pujol (IGTP), Barcelona, Spain; CIBER Epidemiologia y Salud Pública (CIBERESP), Barcelona, Spain; Departament de Salut, Centre d’Estudis Epidemiològics sobre les Infeccions de Transmissió Sexual i Sida de Catalunya (CEEISCAT), Barcelona, Spain; Institut d'Investigació Germans Trias i Pujol (IGTP), Barcelona, Spain; CIBER Epidemiologia y Salud Pública (CIBERESP), Barcelona, Spain; Departament de Salut, Centre d’Estudis Epidemiològics sobre les Infeccions de Transmissió Sexual i Sida de Catalunya (CEEISCAT), Barcelona, Spain; Institut d'Investigació Germans Trias i Pujol (IGTP), Barcelona, Spain; Departament de Salut, Centre d’Estudis Epidemiològics sobre les Infeccions de Transmissió Sexual i Sida de Catalunya (CEEISCAT), Barcelona, Spain; Institut d'Investigació Germans Trias i Pujol (IGTP), Barcelona, Spain; Infectious Disease Unit, Hospital Universitari Germans Trias i Pujol, Badalona, Spain; Department of Infectious Diseases, HIV Infection Unit, Hospital de la Santa Creu i Sant Pau, Barcelona, Spain; Department of Infectious Diseases, Hospital Universitari de Bellvitge, Institute of Biomedical Research of Bellvitge (IDIBELL), L'Hospitalet de Llobregat, Spain; Department of Internal Medicine, Hospital de Son Llàtzer, Palma, Spain; Hospital Universitario Son Espases, Health Research Institute of the Balearic Islands (IdISBa), Palma, Spain; Department of Infectious Diseases, Hospital Universitari Vall d’Hebron, Vall d'Hebron Research Institute (VHIR), Barcelona, Spain; Hospital Clínic-Institut d’Investigacions Biomèdiques August Pi i Sunyer, University of Barcelona, Barcelona, Spain; Department of Internal Medicine-Infectious Diseases, Hospital del Mar, Barcelona, Spain; Internal Medicine and Infectious Disease Service, Hospital Verge de la Cinta, Tortosa, Spain; Departament de Salut, Centre d’Estudis Epidemiològics sobre les Infeccions de Transmissió Sexual i Sida de Catalunya (CEEISCAT), Barcelona, Spain; Department of Infectious Diseases, Odense University Hospital, Odense, Denmark; Hospital Clínic-Institut d’Investigacions Biomèdiques August Pi i Sunyer, University of Barcelona, Barcelona, Spain; CIBERINFEC, Instituto de Salud Carlos III, Madrid, Spain; Departament de Salut, Centre d’Estudis Epidemiològics sobre les Infeccions de Transmissió Sexual i Sida de Catalunya (CEEISCAT), Barcelona, Spain; Institut d'Investigació Germans Trias i Pujol (IGTP), Barcelona, Spain; CIBER Epidemiologia y Salud Pública (CIBERESP), Barcelona, Spain; Departament de Pediatria, d’Obstetrícia i Ginecologia i de Medicina Preventiva i de Salut Publica, Universitat Autònoma de Barcelona, Bellaterra, Spain; Departament de Salut, Centre d’Estudis Epidemiològics sobre les Infeccions de Transmissió Sexual i Sida de Catalunya (CEEISCAT), Barcelona, Spain

**Keywords:** AIDS, antiretroviral therapy, HIV, mortality, non-AIDS cancers

## Abstract

**Background:**

Effective antiretroviral therapy (ART) has substantially reduced acquired immunodeficiency syndrome (AIDS)-related deaths, shifting the focus to non-AIDS conditions in people living with human immunodeficiency virus (HIV) (PLWH). We examined mortality trends and predictors of AIDS- and non-AIDS mortality in the Population HIV Cohort from Catalonia and Balearic Islands (PISCIS) cohort of PLWH from 1998 to 2020.

**Methods:**

We used a modified Coding Causes of Death in HIV protocol, which has been widely adopted by various HIV cohorts to classify mortality causes. We applied standardized mortality rates (SMR) to compare with the general population and used competing risks models to determine AIDS-related and non-AIDS-related mortality predictors.

**Results:**

Among 30 394 PLWH (81.5% male, median age at death 47.3), crude mortality was 14.2 per 1000 person-years. All-cause standardized mortality rates dropped from 9.6 (95% confidence interval [CI], 8.45–10.90) in 1998 through 2003 to 3.33 (95% CI, 3.14–3.53) in 2015 through 2020, *P* for trend = .0001. Major causes were AIDS, non-AIDS cancers, cardiovascular disease, AIDS-defining cancers, viral hepatitis, and nonhepatitis liver disease. Predictors for AIDS-related mortality included being aged ≥40 years, not being a man who have sex with men, history of AIDS-defining illnesses, CD4 < 200 cells/µL, ≥2 comorbidities, and nonreceipt of ART. Non-AIDS mortality increased with age, injection drug use, heterosexual men, socioeconomic deprivation, CD4 200 to 349 cells/µL, nonreceipt of ART, and comorbidities, but migrants had lower risk (adjusted hazard risk, 0.69 [95% CI, .57–.83]).

**Conclusions:**

Mortality rates among PLWH have significantly decreased over the past 2 decades, with a notable shift toward non–AIDS-related causes. Continuous monitoring and effective management of these non-AIDS conditions are essential to enhance overall health outcomes.

## Teaser

We analyzed acquired immunodeficiency syndrome (AIDS) and non-AIDS deaths among people with human immunodeficiency virus in Catalonia, Spain, from 1998 through 2020. Leading causes were AIDS, cancers, and cardiovascular disease. AIDS-related causes decreased, whereas non-AIDS causes increased highlighting the need for continuous monitoring of these conditions.

Despite the notable progress made in reducing morbidity and mortality of people living with human immunodeficiency virus (HIV) (PLWH), the global impact of the virus remains significant. The introduction of safe, tolerable, and efficacious antiretroviral therapy (ART) [1, 2], coupled with strategies such as test-and-treat and universal ART initiation [3], and the development of direct-acting antivirals for hepatitis C virus (HCV) [4], have transformed the landscape of HIV care making the life expectancy of PLWH similar to the general population [5]. These advancements have averted nearly 21 million acquired immunodeficiency syndrome (AIDS)-related deaths between 1996 and 2022 [6] and changed the patterns in the causes of mortality in this population.

Yet, in 2022 alone, an estimated 630 000 (480 000–880 000) AIDS-related deaths occurred globally [6]. Increased mortality among PLWH has been associated with older age at seroconversion, longer duration of HIV infection, ART failure, suboptimal adherence to treatment, late diagnosis, and HIV-related risk behaviors such as injection drug use [7–9].

In Spain, HIV remains a key challenge for health authorities despite the wide availability and accessibility to ART. It is estimated that there are approximately 150 000 PLWH in the country representing about 0.4% of the general population [6]. A total of 2786 new diagnoses were reported to the Spanish System of Information on New Diagnoses of HIV Infection as of June 2022, which represents an incidence rate of 5.89 per 100 000 inhabitants [10]. Compared with other Western European countries, these figures are higher. A study from Spain reported that between 1999–2003 and 2014–2018, overall mortality among PLWH decreased from 33.5 to 20.7 per 1000 person-years, with AIDS-related deaths dropping from 64% to 35%, although HIV-related mortality remained about 7 times higher than in the general population during 2018 [11]. Aside the relatively smaller sample size of the study, the description of causes of death was not exhaustive [11].

Monitoring the evolution of causes of mortality among PLWH capacitates strategic planning and implementation of interventions that enhance patient care, improve management of comorbid conditions, and prevent avoidable deaths. Although several HIV cohorts have described the mortality rates over time, the changes in the patterns of mortality causes and the contribution of AIDS- and non-AIDS conditions to HIV mortality are inadequately described with limited information on potential differential predictors of AIDS-related and non–AIDS-related mortality.

We described the mortality rates, changing patterns, and causes of death among PLWH in Catalonia and the Balearic Islands, Spain. We additionally investigated the predictors of AIDS-related and non–AIDS-related mortality.

## METHODS

### Study Design, Participants, and Data Sources

We conducted a retrospective cohort study using the Population HIV Cohort from Catalonia and Balearic Islands (PISCIS), Spain, from 1998 to 2020. The cohort design has been described elsewhere [12]. Briefly, PISCIS is a multicenter, prospective, observational study that has continuously enrolled individuals aged ≥16 years living with HIV who receive care at 17 collaborating hospitals in Catalonia and 2 in the Balearic Islands since its inception in 1998. We restricted the current analyses to participants who were in clinical follow-up during the study period (with at least 1 visit within a 12-month period) to avoid the competing risk of loss to follow-up.

Mortality data were sourced from the collaborating hospitals within the PISCIS cohort. Clinicians routinely report causes of death as part of the cohort’s surveillance protocol. To ensure the accuracy and completeness of the mortality data, we conducted triangulation with data obtained from 2 external sources: the National Institute of Statistics and the Data Analytics Program for Health Research and Innovation in Catalonia (PADRIS).

Mortality data for the general population of Spain stratified by sex and age were obtained from the National Institute of Statistics [13] by year of death.

### Categorization of Causes of Death

We used a modified Coding Causes of Death in HIV (CoDe) protocol [14] to classify causes of death independently by 2 clinicians using the International Classification of Diseases, 9th and 10th revisions (ICD-9 and ICD-10). A third clinician was invited to resolve disputes when necessary. The CoDe protocol leverage both death certificates and clinical markers and has been widely adopted by various HIV cohorts to classify causes of death [14]. We grouped causes of death under 17 categories based on the CoDe protocol and further classified them into AIDS-related and non–AIDS-related causes. The 17 categories of causes of death are listed in Supplementary Table 1.

### Statistical Analysis

We used descriptive statistics to summarize baseline characteristics and outcomes. Follow-up time was from 1 January 1998, or at cohort entry until death or 31 December 2020, whichever came first.

We calculated crude all-cause mortality rates across epidemiological and clinical groups over time. Crude mortality rates (CMRs) were determined by dividing the total number of deaths by the total number of person-years of follow-up and multiplying by 1000 to obtain rates per 1000 person-years.

To compare the mortality in PLWH versus the general population, we calculated standardized mortality ratios (SMRs) according to the predefined calendar periods (1998–2003, 2004–2008, 2009–2014, 2015–2020), stratified by sex. We calculated 95% confidence intervals (CIs) using Poisson distribution. We chose calendar years based on the evolving epidemiology of the HIV epidemic in Catalonia over the years: 1998 through 2003 represents the early years of combined ART; in 2004 through 2009, infection through injected drug use peaked and subsequently from 2010 men who have sex with men (MSM) became the most common route of transmission; in 2015, second-generation direct-acting antivirals for the treatment of HCV and immediate ART initiation became widely implemented. Counts were used to quantify the proportions of mortality causes over time.

To assess the associations between potential risk factors and mortality from AIDS-related or non-AIDS causes, we used unadjusted and adjusted competing risk models providing hazard ratios with 95% CI. The multivariable analysis was adjusted for sex at birth, age at cohort entry, country of origin, HIV transmission risk group, socioeconomic deprivation, HIV viral load and CD4 cell count at cohort entry, calendar year of HIV diagnosis, reception of ART, history of AIDS-defining illness, and comorbidities. We checked collinearity by calculating variance inflation factors in the covariate list in the primary adjusted model.

In secondary analyses, we performed multivariate imputation by chained equations to account for missing data on CD4 cell count, HIV RNA viral load, country of origin, and socioeconomic status to examine differences in CMRs (Supplementary Table 2). Statistical significance was set at a *P* value of <.05 (2-sided). We did all analyses in R (version 4.1.3).

### Ethics Declaration

The PISCIS cohort study received ethical approval from the Ethics Committee of the Germans Trias i Pujol University Hospital, Badalona, Spain (EO-11-108). Patient-level information obtained from PADRIS was anonymized and deidentified before the analyses. This study adheres to the Strengthening the Reporting of Observational Studies in Epidemiology guidelines for transparent and accurate reporting of observational studies. The planning, conduct, and reporting of the study were carried out in accordance with the principles outlined in the Declaration of Helsinki, as revised in 2013.

## RESULTS

From 1 January 1998 to 31 December 2020, our cohort included 30 394 PLWH, contributing to a cumulative follow-up time of 288 780 person-years. The median follow-up period per individual was 8.7 years (interquartile range [IQR]: 3.6–14.4). A description of baseline cohort characteristics is provided in Table 1.

**Table 1.** Cohort Characteristics at Baseline and Crude Mortality Rates

	Overall Cohort, *N* (%)	Dead, *N* (%)	*P* Value	PY (×1000)	CMR per 1000 PY (95% CI)
Age at cohort entry (y)	…	…	<.0001	…	…
16–29	8501 (28.0)	614 (15.0)	…	83 133.4	7.39 (7.37–7.40)
30–39	12 412 (40.8)	1631 (39.8)	…	123 179.6	13.24 (13.22–13.26)
40–49	6533 (21.5)	1089 (26.6)	…	58 541.6	18.60 (18.57–18.64)
50–64	2548 (8.4)	564 (13.8)	…	21 064.7	26.77 (26.70–26.84)
≥ 65	400 (1.3)	204 (5.0)	…	2861.4	71.29 (70.98–71.60)
Age at cohort entry, median (IQR), y	35.2 (29.3–42.0)	38.2 (32.9–47.0)	<.0001	…	…
Age at death or last contact, median (IQR), y	46.3 (38.2–54.7)	47.3 (40.5–54.9)	<.0001	…	…
Sex	…	…	.003	…	…
Male	24 755 (81.5)	3272 (79.8)	…	224 858.7	14.55 (14.54–14.57)
Female	5634 (18.5)	829 (20.21)	…	63 906.3	12.97 (12.94–13.00)
Missing	5 (0.02)	1 (0.02)	…	15.6	64.13 (60.16–68.11)
Region of origin	…	…	<.0001	…	…
Spanish	18 124 (59.6)	3404 (83.0)	…	202 162.4	16.84 (16.82–16.86)
Non-Spanish	11 525 (37.9)	496 (12.1)	…	82 986.6	5.98 (5.96–5.99)
Missing	745 (2.5)	202 (4.9)	…	3631.6	55.62 (55.38–55.87)
Socioeconomic deprivation	…	…	<.0001	…	…
Least socioeconomic deprivation	11 834 (38.9)	1239 (30.2)	…	108 241.7	11.45 (11.43–11.47)
Mild socioeconomic deprivation	4719 (15.5)	758 (18.5)	…	50 874.9	14.9 (14.87–14.93)
Moderate/severe socioeconomic deprivation	7311 (24.1)	1295 (31.6)	…	78 452.9	16.51 (16.48–16.54)
Missing	6530 (21.5)	810 (19.8)	…	51 211.0	15.82 (15.78–15.85)
HIV transmission route	…	…	<.0001	…	…
MSM	14 105 (46.4)	610 (14.9)	…	115 933.9	5.26 (5.25–5.27)
PWID	5660 (18.6)	2002 (48.8)	…	67 541.9	29.64 (29.60–29.68)
Male heterosexual	4313 (14.2)	743 (18.1)	…	41 886.4	17.74 (17.70–17.78)
Women infected through sex	3832 (12.6)	344 (8.4)	…	43 375.2	7.93 (7.90–7.96)
Other	918 (3.0)	130 (3.2)	…	7960.9	16.33 (16.24–16.42)
Missing	1566 (5.2)	273 (6.7)	…	12 082.2	22.6 (22.51–22.68)
Period of HIV diagnosis	…	…	<.0001	…	…
1981–1997	5306 (17.5)	1653 (40.3)	…	72 892.1	22.68 (22.64–22.71)
1998–2003	6247 (20.6)	1442 (35.15)	…	86 285.0	16.71 (16.68–16.74)
2004–2008	5890 (19.4)	633 (15.43)	…	62 587.1	10.11 (10.09–10.14)
2009–2014	7239 (23.8)	300 (7.31)	…	51 471.7	5.83 (5.81–5.85)
2015–2020	5712 (18.8)	74 (1.8)	…	15 544.7	4.76 (4.73–4.79)
Years since HIV diagnosis, median (IQR)	10.8 (5.2–17.8)	11.2 (4.8–18.3)	<.0001	…	…
CD4 count at cohort entry, cells/µL	…	…	<.0001	…	…
<200	5693 (18.7)	1195 (29.1)	…	53 686.1	22.26 (22.22–22.3)
200–349	4577 (15.1)	550 (13.4)	…	41 012.5	13.41 (13.38–13.45)
350–499	4392 (14.5)	361 (8.8)	…	38 164.2	9.46 (9.43–9.49)
≥500	7932 (26.1)	663 (16.2)	…	65 372.2	10.14 (10.12–10.17)
Missing	7800 (25.7)	1333 (32.5)	…	90 545.5	14.72 (14.70–14.75)
CD4 count (cells/µL), median (IQR)	383.5 (198.0–598.0)	244.0 (95.0–480.0)	<.0001	…	…
HIV-RNA viral load at cohort entry	…	…	<.0001	…	…
Detectable	2356 (7.8)	91 (2.2)	…	9781.5	9.30 (9.24–9.36)
Undetectable	19 994 (65.8)	2574 (62.8)	…	184 620.6	13.94 (13.93–13.96)
Missing	8044 (26.5)	1437 (35.0)	…	94 378.4	15.23 (15.2–15.25)
History of AIDS-defining illness	…	…	<.0001	…	…
No	24 836 (81.7)	2497 (60.9)	…	226 353.2	11.03 (11.02–11.05)
Yes	5558 (18.3)	1605 (39.1)	…	62 427.3	25.71 (25.67–25.75)
ART at death or last contact	…	…	<.0001	…	…
Yes	26 828 (88.3)	2834 (69.1)	…	269 998.7	10.50 (10.48–10.51)
No	3566 (11.7)	1268 (30.9)	…	18 781.8	67.51 (67.39–67.63)
Years on ART, median (IQR)	8.0 (3.7–13.4)	6.0 (2.1–11.0)	<.0001	…	…
Comorbidities	…	…	…	…	…
Myocardial infarction	608 (2.0)	119 (2.9)	<.0001	8857.4	13.44 (13.36–13.51)
Congestive heart failure	798 (2.6)	205 (5.0)	<.0001	11 189.7	18.32 (18.24–18.40)
Peripheral vascular disease	637 (2.1)	151 (3.7)	.0001	9435.4	16.00 (15.92–16.08)
Cerebrovascular disease	1072 (3.5)	237 (5.8)	<.0001	14 184.1	16.71 (16.64–16.78)
Dementia	220 (0.7)	80 (2.0)	<.0001	2994.2	26.72 (26.53–26.90)
Chronic pulmonary disease	4552 (15.0)	707 (17.2)	<.0001	58 099.3	12.17 (12.14; 12.20)
Rheumatoid disease	165 (0.5)	18 (0.4)	>.99	2137.2	8.42 (8.30–8.55)
Peptic ulcer disease	371 (1.2)	62 (1.5)	.003	4731.6	13.10 (13.00–13.21)
Mild liver disease	5937 (19.5)	1324 (32.3)	<.0001	79 006.5	16.76 (16.73–16.79)
Diabetes without chronic complications	1587 (5.2)	291 (7.1)	<.0001	21 866.0	13.31 (13.26–13.36)
Diabetes with chronic complications	282 (0.9)	77 (1.9)	<.0001	3976.0	19.37 (19.23–19.50)
Hemiplegia or paraplegia	410 (1.4)	107 (2.6)	<.0001	5091.6	21.02 (20.89–21.14)
Renal disease	1977 (6.5)	268 (6.5)	<.0001	24 287.8	11.03 (10.99–11.08)
Cancer (any malignancy)	2674 (8.8)	844 (20.6)	<.0001	32 144.4	26.26 (26.20–26.31)
Moderate or severe liver disease	582 (1.9)	293 (7.1)	<.0001	80 078.0	36.59 (36.46–36.72)
Metastatic solid tumor	566 (1.9)	339 (8.3)	<.0001	7032.4	48.21 (48.04–48.37)
Number of comorbidities, median (IQR)	0 (0.0–1.0)	2.0 (1.0–3.0)	<.0001	…	…
Number of comorbidities	…	…	<.0001	…	…
0	12 428 (51.0)	634 (23.6)	…	107 637.9	5.89 (5.88–5.90)
1	6342 (26.0)	657 (24.5)	…	71 102.5	9.24 (9.22–9.26)
2	2975 (12.2)	530 (19.8)	…	37 605.7	14.09 (14.06–14.13)
≥3	2646 (10.9)	861 (32.1)	…	37 995.8	22.66 (22.61–22.71)

Abbreviations: ART, antiretroviral therapy, undetectable HIV-RNA was defined as ≤50 copies/mL; CI, confidence interval; CMR, crude mortality rate; IQR, interquartile range; MSM, men who have sex with men; PWID, people who inject drugs; PY, person-years.

At cohort entry, the median age of the overall cohort was 35.2 years (IQR: 29.3–42.0), which increased to 46.3 years (IQR: 38.2–54.7) at the time of death or last contact. Most of cohort participants were male (81.5%) and of Spanish origin (59.6%). The predominant HIV transmission risk group was MSM, accounting for 46.4% of the cohort, followed by people who inject drugs (PWID) at 18.6%. Among patients who died, the median time interval from HIV diagnosis to death was 11.2 years (IQR: 4.8–18.3). At cohort entry, participants had a median CD4 cell count of 383.5 cells/µL (IQR: 198.0–598.0), with 7.8% of those with viral load measurements having a detectable HIV RNA viral load. A majority (88.3%) was receiving ART at the time of death or last contact, and 62.9% of PLWH in our cohort presented no comorbidities at baseline (Table 1). The characteristics of deaths in the cohort according to calendar periods are presented in Supplementary Table 3.

During the 23 years of observation, 4102 PLWH (13.5%) died representing an all-cause CMR of 14.4 per 1000 person-years (95% CI, 13.9–14.8) (Table 1). Notably, we observed a decline in overall all-cause SMRs. The SMR was 9.60 (95% CI, 8.45–10.90) per 1000 person-years in 1998–2003 and declined to 7.92 (7.39–8.49) in 2004–2008, further to 5.55 (5.23–5.88) in 2009–2014. In 2015–2020, the SMR was 3.33 (95% CI, 3.14–3.53) per 1000 person-years, *P* for trend <.0001. The SMRs across the calendar years studied were consistently higher in women compared with men (Table 2).

**Table 2.** Crude and Standardized Mortality Rates by Sex in Different Calendar Periods

	Overall Cohort			Men			Women		
Period	CMR	ASMR	95% CI	CMR	ASMR	95% CI	CMR	ASMR	95% CI
1998–2003	17.32	9.6	8.45–10.9	18.86	7.08	6.14–8.17	13.18	13.58	10.27–17.97
2004–2008	18.87	7.92	7.39–8.49	20.64	5.96	5.52–6.44	13.8	11.27	9.6–13.22
2009–2014	14.37	5.55	5.23–5.88	14.79	4.13	3.86–4.41	13.04	8.58	7.56–9.74
2015–2020	10.93	3.33	3.14–3.53	10.62	2.5	2.34–2.67	12.15	5.02	4.44–5.68
*P* for trend	**…**	<.0001	**…**	**…**	<.0001	**…**	**…**	<.0001	**…**

Abbreviations: ASMR, age-standardized mortality rate; CI, confidence interval; CMR, crude mortality rate.

The cause of death could not be classified or was unknown for 288 (7.0%) of 4102 deaths. The key causes of death were AIDS accounting for 1115 deaths (including 213 AIDS-defining cancers), non-AIDS cancers (705 deaths), and cardiovascular disease (CVD; 377 deaths). Viral hepatitis accounted for 209 of deaths, and noncancer nonhepatitis liver disease accounted for 208 deaths. Lung cancers (33.7%) and liver cancers (15.6%) were the highest causes of non-AIDS cancer mortality, whereas non-Hodgkin lymphoma (59.3%) and Kaposi sarcoma (18.6%) were the highest causes of AIDS-defining cancers (Supplementary Figure 1).

**Figure 1.**
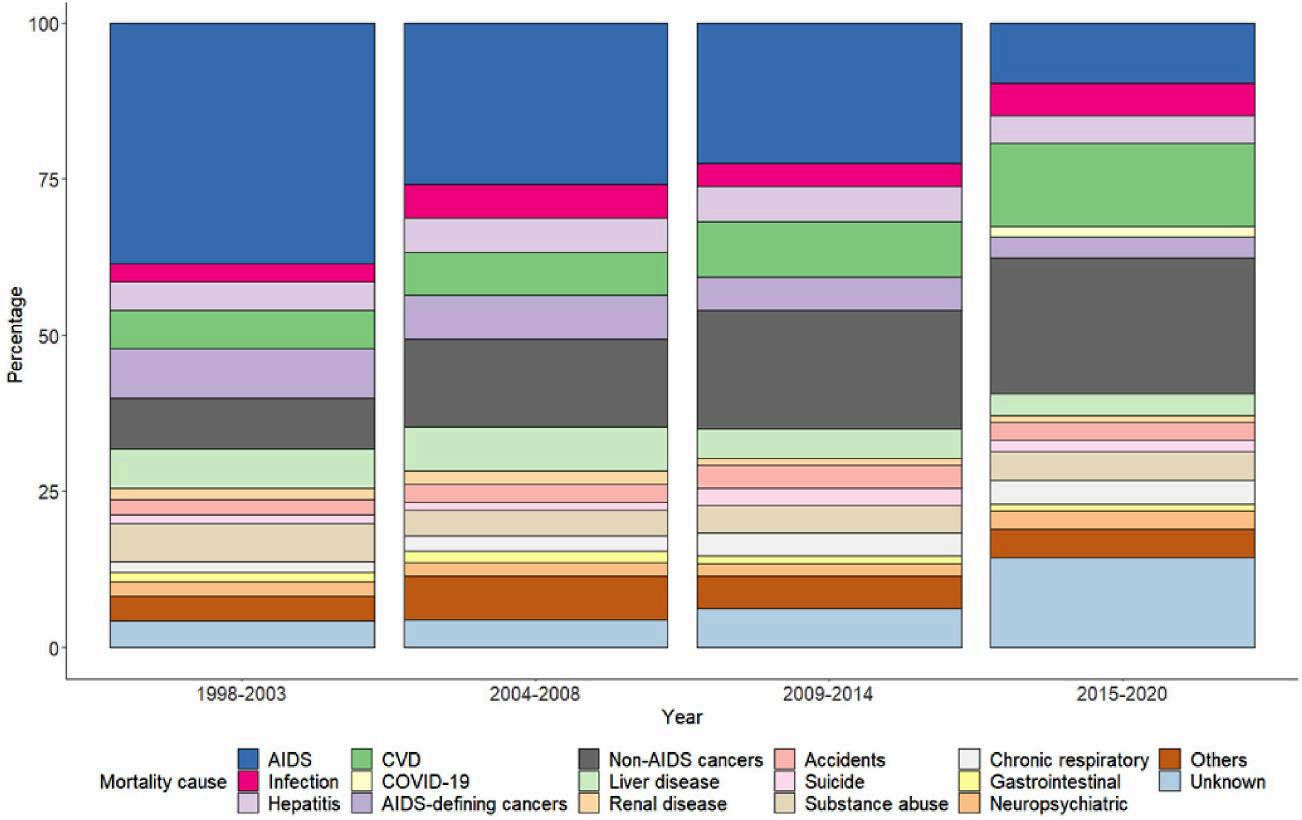
Causes of death among people living with HIV in Catalonia and the Balearic Islands, Spain, 1998–2020, by calendar periods. Abbreviations: CVD, cardiovascular disease; COVID-19, coronavirus disease 2019.

**Figure 2.**
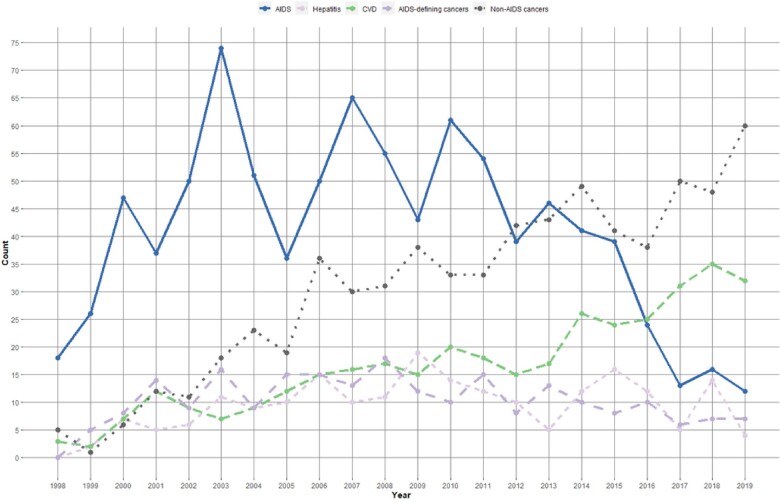
Number of deaths to key AIDS-related and non-AIDS causes among people living with HIV in Catalonia and the Balearic Islands, Spain, 1998–2020. Abbreviations: CVD, cardiovascular disease.

There was a significant reduction in the proportion of AIDS-related mortality, declining from 38.5% during the period of 1998 through 2003 to 9.8% during 2015 through 2020 (*P* < .0001). A similar decline was observed for AIDS-defining cancers, decreasing from 7.9% in 1998–2003 to 3.4% in 2015–2020 (*P* < .0001). In contrast, non–AIDS-related cancers increased, rising from 8.1% in 1998 through 2003 to 22.1% in 2015 through 2020 (*P* < .0001). Similarly, mortality attributed to CVD, surged from 6.1% during 1998 through 2003 to 13.5% in 2015 through 2020 (*P* < .0001). The median ages of PLWH who died from cancers and CVD increased across the calendar periods (Supplementary Table 1). Deaths attributable to viral hepatitis (hepatitis B and C viruses) remained relatively steady during the study period. Specifically, the proportion of hepatitis-related mortality stood at 4.7% in 1998 through 2003, increased to 5.5% in 2004 through 2008, reached 5.7% in 2009 through 2014, and decreased to 4.6% in 2015 through 2020 (*P* = .763) (Figures 1 and 2, Supplementary Table 4). The causes of death by years is depicted in Supplementary Figure 2, Supplementary Table 5. We further classified causes of death according to years since enrollment into the cohort in Supplementary Figure 3.

Age 40 years and older was associated with a significant increase in AIDS-related mortality. Compared with MSM, we observed an increased AIDS mortality risk among PWID (adjusted hazard risk, 2.77 [95% CI, 1.97–3.90]), male heterosexuals (1.66 [1.19–2.31]), and women infected through sex (1.75 [1.01–3.03]). The risk of AIDS-related mortality was higher among PLWH with CD4 < 200 cells/µL (1.53 [1.16–2.02]), those with a history of an AIDS-defining illness (4.22 [3.35–5.32]), and those with 2 comorbidities (1.38 [1.02–1.98]) compared with those without comorbidities. Nonreception of ART elevated the risk of AIDS-defining illness by 7-fold (7.60 [5.76–10.04]) (Table 3).

**Table 3.** Competing Risk Models for AIDS-related and Non–AIDS-related Mortality

	Non-AIDS Model (Univariable)		Non-AIDS Model (Multivariable)		AIDS Model (Univariable)		AIDS Model (Multivariable)	
	HR (95% CI)	*P* Value	HR (95% CI)	*P* Value	aHR (95% CI)	*P* Value	aHR (95% CI)	*P* Value
Age at recruitment, y								
16–29 (ref)	1	…	1	…	1	…	1	…
30–39	1.78 (1.59–1.99)	<.0001	1.55 (1.25–1.92)	<.0001	1.97 (1.67–2.32)	<.0001	1.01 (.72–1.40)	.973
40–49	2.74 (2.43–3.09)	<.0001	2.51 (2.01–3.14)	<.0001	2.50 (2.09–2.98)	<.0001	1.61 (1.14–2.27)	.007
50–64	4.01 (3.49–4.60)	<.0001	4.22 (3.27–5.45)	<.0001	3.44 (2.80–4.24)	<.0001	2.26 (1.51–3.40)	<.0001
≥65	10.74 (8.84–13.05)	<.0001	8.92 (6.19–12.87)	<.0001	9.95 (7.54–13.14)	<.0001	4.27 (2.26–8.06)	<.0001
Sex								
Male (ref)	1	…	1	…	1	…	1	…
Female	.79 (.72–.87)	<.0001	.97 (.78–1.2)	.765	1.06 (.93–1.21)	.37	.80 (.53–1.20)	.283
Region of origin								
Spanish (ref)	1	…	1	…	1	…	1	…
Non-Spanish	.36 (.32–.41)	<.0001	.69 (.57–.83)	<.0001	.36 (.3–.42)	<.0001	.75 (.56–1.00)	.052
HIV transmission route								
MSM (ref)	1	…	1	…	1	…	1	…
PWID	5.58 (4.99–6.24)	<.0001	3.38 (2.77–4.12)	<.0001	5.47 (4.66–6.42)	<.0001	2.77 (1.97–3.9)	<.0001
Male heterosexual	3.27 (2.87–3.73)	<.0001	1.98 (1.64–2.40)	<.0001	3.57 (2.97–4.30)	<.0001	1.66 (1.19–2.31)	.003
Women infected through sex	1.35 (1.14–1.59)	<.0001	1.06 (.76–1.47)	.736	1.83 (1.46–2.28)	<.0001	1.75 (1.01–3.03)	.048
Other	2.86 (2.26–3.62)	<.0001	1.31 (.85–2.02)	.214	3.50 (2.55–4.8)	<.0001	3.26 (1.97–5.39)	<.0001
Socioeconomic deprivation								
Least socioeconomic deprivation (ref)	1	…	1	…	1	…	1	…
Mild socioeconomic deprivation	1.26 (1.13–1.40)	<.0001	.92 (.78–1.09)	.327	1.34 (1.13–1.57)	<.0001	.82 (.61–1.09)	.167
Moderate/severe socioeconomic deprivation	1.45 (1.32–1.59)	<.0001	1.16 (1.01–1.34)	.038	1.35 (1.17–1.56)	<.0001	1.00 (.79–1.27)	.994
Period of HIV diagnosis								
1998–2003 (ref)	1	…	1	…	1	…	1	…
2004–2008	.47 (.42–.52)	<.0001	1.17 (.96–1.44)	.128	.42 (.36–.49)	<.0001	.95 (.68–1.33)	.774
2009–2014	.29 (.25–.34)	<.0001	1.15 (.89–1.48)	.28	.19 (.15–.23)	<.0001	.86 (.57–1.29)	.471
2015–2020	.25 (.19–.33)	<.0001	1.53 (1.02–2.28)	.04	.09 (.06–.14)	<.0001	.46 (.21–1.03)	.06
CD4 count at cohort entry, cells/µL								
≥500 (ref)	1	…	1	…	1	…	1	…
350–499	1.01 (.87–1.18)	.861	1.05 (.86–1.28)	.629	.75 (.58–.97)	.026	.80 (.54–1.18)	.26
200–349	1.41 (1.23–1.61)	<.0001	1.27 (1.06–1.51)	.009	1.12 (.90–1.40)	.314	.98 (.69–1.39)	.92
<200	1.78 (1.59–2.01)	<.0001	1.07 (.90–1.26)	.454	3.23 (2.74–3.81)	<.0001	1.53 (1.16–2.02)	.003
HIV-RNA viral load at cohort entry								
Undetectable (ref)	1	…	1	…	1	…	1	…
Detectable	1.28 (1.01–1.64)	.044	1.35 (.97–1.87)	.073	2.36 (1.56–3.58)	<.0001	1.42 (.78–2.57)	.247
History of AIDS-defining illness								
No (ref)	1	…	1	…	1	…	1	…
Yes	1.57 (1.45–1.07)	<.0001	1.15 (1.00–1.32)	.054	4.89 (4.39–5.46)	<.0001	4.22 (3.35–5.32)	<.0001
ART at death or last contact								
Yes (ref)	1	…	1	…	1	…	1	…
No	5.81 (5.34–6.32)	<.0001	5.78 (4.82–6.92)	<.0001	8.59 (7.68–9.60)	<.0001	7.60 (5.76–10.04)	<.0001
Number of comorbidities								
0 (ref)	1	…	1	…	1	…	1	…
1	1.57 (1.37–1.79)	<.0001	1.24 (1.03–1.50)	.026	1.38 (1.15–1.66)	<.0001	1.04 (.78–1.40)	.78
2	2.38 (2.06–2.74)	<.0001	1.56 (1.27–1.91)	<.0001	1.93 (1.57–2.36)	<.0001	1.38 (1.02–1.89)	.039
≥3	4.25 (3.76–4.81)	<.0001	2.43 (2.01–2.95)	<.0001	1.92 (1.57–2.36)	<.0001	1.19 (.86–1.65)	.298

Abbreviations: ART, antiretroviral therapy; aHR, adjusted hazard risk; HR; unadjusted hazard risk; IQR, interquartile range; MSM, men who have sex with men; PWID, people who inject drugs; ref, reference group in multivariable analysis.

Undetectable HIV-RNA was defined as ≤50 copies/mL. Model adjusted for sex, age, region of origin, socioeconomic deprivation, HIV transmission group, presence of an AIDS-defining illness, backbone ART, plasma HIV-RNA viral load (categorized detectable and undetectable), CD4 cell count (categorized <200, 200–349, 349–499, >500 cells/µL), and comorbidities.

Expectedly, the risk of non–AIDS-related mortality increased with age. Compared with MSM, we found an elevated risk of non-AIDS mortality among PWID (adjusted hazard ratio, 3.38 [95% CI, 2.77–4.12]) and male heterosexuals (1.98 [1.64–2.40]). Additionally, PLWH with moderate to severe socioeconomic deprivation (1.16 [1.01–1.34]) and those diagnosed in 2015 through 2020 (1.53 [1.02–2.28]) had an elevated risk of non-AIDS mortality. CD4 cell count of 200 to 349 cells/µL at cohort entry (1.27 [1.06–1.51]) was associated with a high risk but not higher or lower values. We found an almost 6-fold increased risk of non-AIDS mortality in PLWH not receiving ART (5.78 [4.82–6.92]). An increasing number of comorbidities was associated with an increasing risk of non-AIDS mortality. On the other hand, migrants experienced a reduced risk of non–AIDS-related mortality (0.69 [0.57–0.83]) (Table 3).

## DISCUSSION

We present a retrospective analysis spanning 23 years of data from a large prospective cohort of PLWH to assess the evolving trends in AIDS- and non–AIDS-related mortality and their predictors. Our findings show a changing landscape of mortality among PLWH over 2 decades.

We observed a substantial decline in overall mortality among PLWH in Catalonia and the Balearic Islands from 1998 to 2020, consistent with prior studies [1, 2]. However, despite the significant decline, mortality among PLWH remains more than 3 times higher than that in the general Spanish population in 2015 through 2020. Delays in HIV testing, linkage to care, and treatment initiation continue to contribute to this disparity, like other European countries, highlighting the obstinate unmet need of universal and earlier diagnosis of occult HIV infection and some ongoing health disparities. Lifestyle factors such as smoking, alcohol consumption, and recreational drug use are frequently observed among PLWH and may contribute to higher mortality rates compared with the general population [15, 16]. Furthermore, the history of injection drug use among PLWH, particularly during the 2004 through 2009 period in Catalonia and Balearic Islands, is closely associated with increased rates of liver-related illnesses and hepatitis C infections [17]. These factors likely play a significant role in the elevated mortality rates observed among PLWH. Although all-cause crude mortality was slightly higher in men than women, the SMR contradictorily appears higher in women, likely because of relatively elevated mortality rates among younger men in the Spanish general population [13]. The elevated SMR in women may also stem from the higher likelihood of delayed diagnosis among women as reported in the World Health Organization European Region [18], with lower CD4 cell counts and an increased risk of opportunistic infections.

Compared with other European cohorts of PLWH, our study reports a lower proportion of AIDS-related deaths. The 27.2% AIDS-related deaths (including AIDS-defining cancers) is lower than the reported in earlier studies in Catalonia, Spain (1997–2004, 40.4%) [19], Salerno, Italy (1998–2009, 40.4%) [20], Denmark (1995–2005, 40.4%) [21], and the 41.9% from 16 cohorts from Europe and North America (1996–2009) [22]. The observed AIDS-related mortality was however similar to the Data collection on Adverse events of Anti-HIV Drugs study that reported an AIDS-related mortality of 28.7% between 1999 and 2011 [23]. The consistent decline in AIDS-related mortality across these studies underscores the positive impact of enhanced effectiveness and improved access to ART. Differences in mortality trends and the proportion of AIDS-related mortality among cohorts may arise from variations in sociodemographic characteristics, underlying clinical features, access to ART, timing of ART initiation with immediate ART initiation being widely implemented in Spain following the INSIGHT Strategic Timing of AntiRetroviral Treatment trial results [24], and differences in observation periods in various studies.

Contrary to the decline of AIDS-related mortality, our study revealed a steady rise in the mortality attributable to non–AIDS-related causes primarily driven by non-AIDS cancers (22.8%) and CVD (14.0%) as leading causes of death in the 2015 through 2020 period. This finding aligns with recent reports indicating that non-AIDS cancers and CVD are the current leading causes of death in PLWH [25, 26]. Notably, our study shows that the increase in non-AIDS mortality is also a result of the increasing age of the PLWH emphasizing the importance of addressing evolving health challenges in this aging population. Factors such as chronic low-level inflammation in HIV [27], and unhealthy lifestyle behaviors, including smoking, alcohol and drug use, obesity, and physical inactivity could potentially contribute to the increasing mortality from non-AIDS causes among PLWH.

Similar to a US HIV outpatient study [28], our findings did not indicate significant proportional changes in deaths attributed to viral hepatitis (hepatitis B virus and HCV). However, this contrasts with a study from British Columbia, Canada, which reported a significant decrease in hepatitis-related and other liver condition deaths between 1996 and 2012 [29]. In Spain, addressing the burden of viral hepatitis, especially among key populations like PLWH, has become increasingly crucial. Noteworthy initiatives encompass routine testing for hepatitis B and C, provision of antiviral treatments, and ongoing monitoring of liver function and viral load [30].

The strongest predictors of both AIDS and non-AIDS mortality in our study were aged 65 years or older and nonreception of ART. Older age and associated chronic comorbidities are recognized as significant mortality indicators among PLWH and the general population. Overwhelming evidence have demonstrated the impact of ART in reducing morbidity and mortality of PLWH [1, 2] and it is further highlighted in our current study revealing a high mortality risk among PLWH not receiving ART. Despite the accessibility of ART in Spain, approximately 10% of PLWH in our cohort were not on ART at the time of death or last contact. Nonreception of ART is associated with many social determinants of health that have an eventual impact on mortality. These include drug and alcohol addiction, homelessness, severe psychiatric diseases, and violent behavior, among others. Investigating potential barriers to care and devising strategies to reengage these difficult-to-reach PLWH who have disengaged from care is imperative.

Regarding HIV transmission risk groups, the higher risk of mortality among PWID has been widely reported [31]. The elevated risks observed in heterosexuals are similar to findings from a study that assessed all-cause mortality under different transmission categories [32]. Furthermore, the increased risk of mortality among PLWH with CD4 counts <350 cells/µL at cohort entry highlights the urgent need to address the unacceptably high rates of late HIV diagnosis, given its detrimental health impact, including increased mortality.

Interestingly, migrants living with HIV experienced a reduced risk of non–AIDS-related mortality. The finding, however, is similar to a recent international cohort study that reported a non-White racial background as a predictors of lower all-cause mortality [33]. However, the observed lower risk may be influenced by residual confounding, as migrants in our cohort tended to be younger than Spanish individuals (median age in years: 40 [IQR 33–48] vs 49 [IQR 41–56], *P* < .0001). Further studies are warranted to understand the mortality risks among migrants living with HIV.

Our study stands out because of some key strengths. First, although previous research has delved into determinants of all-cause mortality among PLWH, our investigation offers unique insights by identifying distinct predictors of both AIDS-related and non–AIDS-related mortality. Second, our study is a very comprehensive and extensive report on mortality within the PLWH population in Spain. Last, we used an internationally recognized and validated protocol (CoDe) for classifying mortality causes, making our findings more comparable with other large cohort studies and enhancing the reliability of findings.

However, our study had some limitations. Migrants constitute more than one third of the PISCIS cohort and deaths that occur outside of Spain are not accounted for in our analyses. We mitigated this limitation by excluding patients who were not in clinical follow-up for HIV monitoring in the past 12 months. Second, we were unable to ascertain causes of death for some cases, especially in the final years of follow-up because of reporting delays in surveillance data and poor linking from incomplete identifiers. We triangulated multiple databases to reduce the proportion of unknown causes of death. Third, our data set lacks some key variables that have predicted mortality in other studies including alcohol use, smoking, and body mass index. In addition, the socioeconomic deprivation measure that we used in our study is an ecological variable based on an individual's place of residence. The socioeconomic deprivation index takes into account factors such as the proportion of manual workers, residents with a low education level, residents with low income, rate of premature mortality, and rate of avoidable hospitalization of the health areas. A person’s place of residence may indeed not necessarily reflect their socioeconomic deprivation.

In conclusion, our study underscores the substantial reduction in mortality rates among PLWH in Catalonia and the Balearic Islands, Spain, over 2 decades. Despite this, mortality rates remain significantly elevated compared with the general population, even in the recent periods. The shift from AIDS-related to non–AIDS-related causes of death, including non-AIDS cancers and CVD, is notable along the study period, reflecting both the access to ART and the aging of the population. Risk factors for AIDS-related mortality include age ≥40 years, PWID, heterosexual men, women infected through sex, history of AIDS-defining illnesses, CD4 <200 cells/µL at cohort entry, ≥2 comorbidities, and nonreceipt of ART. Non-AIDS mortality risk rises with age ≥30 years, PWID, heterosexual men, socioeconomic deprivation, HIV diagnosis in 2015 through 2020, CD4 200 to 349 cells/µL, nonreceipt of ART, and ≥1 comorbidity. Migrants exhibit a reduced risk, probably related with their younger age. Prioritizing determinants such as late diagnosis is essential. Continuous mortality monitoring informs public health strategies for aging PLWH facing evolving health challenges, highlighting the need for regular screening and effective management of non–AIDS-related illnesses.

## Supplementary Material

ofae132_Supplementary_Data

## Supplementary Data

Supplementary materials are available at *Open Forum Infectious Diseases* online. Consisting of data provided by the authors to benefit the reader, the posted materials are not copyedited and are the sole responsibility of the authors, so questions or comments should be addressed to the corresponding author.

## Notes

***Acknowledgments.*** The authors are grateful to the Public Data Analysis for Health Research and Innovation Program of Catalonia (PADRIS), the Sub-direction of Drug Dependencies, HIV, Sexually Transmitted Diseases, and Viral Hepatitis within the Public Health Secretary of the Catalan Health Department for their valuable support, and all staff of PISCIS collaborating hospitals.

***Role of the funding source.*** This study was funded by Fundació La Marató de TV3, grant number 202117-30-31. The Fundació La Marató de TV3 had no role in the design and conduct of the study; collection, management, analysis, and interpretation of the data; preparation, review, or approval of the manuscript; and decision to submit the manuscript for publication.

***Data sharing.*** The study protocol and statistical code are available from the corresponding authors upon request. The data for this study is available at the Centre for Epidemiological Studies of Sexually Transmitted Diseases and HIV/AIDS in Catalonia (CEEISCAT), the coordinating center of the PISCIS cohort and from each of the collaborating hospitals upon request via https://pisciscohort.org/contacte/.

***Patient consent statement.*** The design of the work has been approved by the Ethics Committee of the Germans Trias i Pujol University Hospital, Badalona, Spain (EO-11-108). Catalan patient data extraction is allowed by of the 203/2015 Decree, of the 15th of September, from the Catalan Health Department. Informed consent is therefore not applicable. PISCIS data is owned by each individual patient, and data is eliminated if requested by the hospital or by a patient. Data is pseudo-anonymized before arriving at the coordinating center, and confidentiality is guaranteed in accordance with the provisions of the Regulation (EU) 2016/679 of the European Parliament and of the Council of 27 April 2016 on the protection of natural persons with regarding the processing of personal data and on the free movement of such data and the new national Organic Law of Protection of Personal Data (3/2018), 5th of December, Data Protection and Digital Rights Act. Patient-level information obtained from PADRIS was anonymised and deidentified prior to the analyses.

***Financial support*.** This study was funded by Fundació La Marató de TV3, grant number 202117-30-31.

## References

[1] Trickey A, May MT, Vehreschild JJ, et al Survival of HIV-positive patients starting antiretroviral therapy between 1996 and 2013: a collaborative analysis of cohort studies. Lancet HIV 2017; 4:e349–56. 28501495 10.1016/S2352-3018(17)30066-8 PMC5555438

[2] Broder S . The development of antiretroviral therapy and its impact on the HIV-1/AIDS pandemic. Antiviral Res 2010; 85:1–18. 20018391 10.1016/j.antiviral.2009.10.002 PMC2815149

[3] Gardner EM, McLees MP, Steiner JF, del Rio C, Burman WJ. The spectrum of engagement in HIV care and its relevance to test-and-treat strategies for prevention of HIV infection. Clin Infect Dis 2011; 52:793–800. 21367734 10.1093/cid/ciq243 PMC3106261

[4] van Santen DK, Sacks-Davis R, Stewart A, et al Treatment as prevention effect of direct-acting antivirals on primary hepatitis C virus incidence: findings from a multinational cohort between 2010 and 2019. EClinicalMedicine 2022; 56:101810. 36618902 10.1016/j.eclinm.2022.101810 PMC9816910

[5] Wandeler G, Johnson LF, Egger M. Trends in life expectancy of HIV-positive adults on ART across the globe: comparisons with general population. Curr Opin HIV AIDS 2016; 11:492–500. 27254748 10.1097/COH.0000000000000298 PMC5055447

[6] Joint United Nations Programme on HIV/AIDS (UNAIDS) . The path that ends AIDS: UNAIDS Global AIDS Update; 2023. Available at: https://thepath.unaids.org/wp-content/themes/unaids2023/assets/files/2023_report.pdf. Accessed 13 November 2023.

[7] Collaboration of Observational HIV Epidemiological Research Europe (COHERE) in EuroCoord, Lewden C, Bouteloup V, et al All-cause mortality in treated HIV-infected adults with CD4 ≥ 500/mm^3^ compared with the general population: evidence from a large European observational cohort collaboration. Int J Epidemiol 2012; 41:433–45. 22493325 10.1093/ije/dyr164

[8] Smith CJ, Ryom L, Weber R, et al Trends in underlying causes of death in people with HIV from 1999 to 2011 (D : A : D): a multicohort collaboration. Lancet 2014; 384:241–8. 25042234 10.1016/S0140-6736(14)60604-8

[9] Helleberg M, Afzal S, Kronborg G, et al Mortality attributable to smoking among HIV-1–infected individuals: a nationwide, population-based cohort study. Clin Infect Dis 2013; 56:727–34. 23254417 10.1093/cid/cis933

[10] Unidad de vigilancia de VIH ITS y hepatitis . Vigilancia Epidemiológica del VIH y sida en España 2021: Sistema de Información sobre Nuevos Diagnósticos de VIH y Registro Nacional de Casos de Sida. Madrid: 2022. Available at: https://www.isciii.es/QueHacemos/Servicios/VigilanciaSaludPublicaRENAVE/EnfermedadesTransmisibles/Documents/VIH/informes de vigilancia VIH y sida anteriores/Informe VIH_SIDA_2022_CCAA.pdf. Accessed 06 November 2023.

[11] Fontela C, Aguinaga A, Moreno-Iribas C, et al Trends and causes of mortality in a population-based cohort of HIV-infected adults in Spain: comparison with the general population. Sci Rep 2020; 10:1–9. 32488053 10.1038/s41598-020-65841-0 PMC7265289

[12] Bruguera A, Nomah D, Moreno-Fornés S, et al Cohort profile: PISCIS, a population-based cohort of people living with HIV in Catalonia and Balearic Islands. Int J Epidemiol 2023; 52:e241. 37403345 10.1093/ije/dyad083

[13] Instituto Nacionalde Estadística (INE) . Indicadores de Mortalidad. 2022. Available at: https://www.ine.es/jaxiT3/Tabla.htm?t=1411. Accessed 06 November 2023.

[14] Kowalska JD, Friis-Møller N, Kirk O, et al The Coding Causes of Death in HIV (CoDe) project: initial results and evaluation of methodology. Epidemiology 2011; 22:516–23. 21522013 10.1097/EDE.0b013e31821b5332

[15] Hasse B, Tarr PE, Marques-Vidal P, et al Strong impact of smoking on multimorbidity and cardiovascular risk among human immunodeficiency virus-infected individuals in comparison with the general population. Open Forum Infect Dis 2015; 2:ofv108. 26284258 10.1093/ofid/ofv108 PMC4536331

[16] Mdodo R, Frazier EL, Dube SR, et al Cigarette smoking prevalence among adults with HIV compared with the general adult population in the United States: cross-sectional surveys. Ann Intern Med 2015; 162:335–44. 25732274 10.7326/M14-0954

[17] Parashar S, Collins AB, Montaner JSG, Hogg RS, Milloy M-J. Reducing rates of preventable HIV/AIDS-associated mortality among people living with HIV who inject drugs. Curr Opin HIV AIDS 2016; 11:507–13. 27254749 10.1097/COH.0000000000000297 PMC5055433

[18] Mårdh O, Quinten C, Kuchukhidze G, et al HIV among women in the WHO European region—epidemiological trends and predictors of late diagnosis, 2009–2018. Eurosurveillance 2019; 24:1900696. 31796153 10.2807/1560-7917.ES.2019.24.48.1900696 PMC6891943

[19] Martinez E, Milinkovic A, Buira E, et al Incidence and causes of death in HIV-infected persons receiving highly active antiretroviral therapy compared with estimates for the general population of similar age and from the same geographical area. HIV Med 2007; 8:251–8. 17461853 10.1111/j.1468-1293.2007.00468.x

[20] Leone S, Gregis G, Quinzan G, et al Causes of death and risk factors among HIV-infected persons in the HAART era: analysis of a large urban cohort. Infection 2011; 39:13–20. 21246246 10.1007/s15010-010-0079-z

[21] Lohse N, Hansen A-BE, Pedersen G, et al Survival of persons with and without HIV infection in Denmark, 1995–2005. Ann Intern Med 2007; 146:87–95. 17227932 10.7326/0003-4819-146-2-200701160-00003

[22] Ingle SM, May MT, Gill MJ, et al Impact of risk factors for specific causes of death in the first and subsequent years of antiretroviral therapy among HIV-infected patients. Clin Infect Dis 2014; 59:287–97. 24771333 10.1093/cid/ciu261 PMC4073781

[23] Smith CJ, Ryom L, Weber R, et al Trends in underlying causes of death in people with HIV from 1999 to 2011 (D:A:D): a multicohort collaboration. Lancet 2014; 384:241–8. 25042234 10.1016/S0140-6736(14)60604-8

[24] INSIGHT START Study Group . Initiation of antiretroviral therapy in early asymptomatic HIV infection. N Engl J Med 2015; 373:795–807. 26192873 10.1056/NEJMoa1506816 PMC4569751

[25] Pettit AC, Giganti MJ, Ingle SM, et al Increased non-AIDS mortality among persons with AIDS-defining events after antiretroviral therapy initiation. J Int AIDS Soc 2018; 21:e25031. 29334197 10.1002/jia2.25031 PMC5810321

[26] Croxford S, Kitching A, Desai S, et al Mortality and causes of death in people diagnosed with HIV in the era of highly active antiretroviral therapy compared with the general population: an analysis of a national observational cohort. Lancet Public Health 2017; 2:e35–46. 29249478 10.1016/S2468-2667(16)30020-2

[27] Brugnaro P, Morelli E, Cattelan F, et al Non-AIDS definings malignancies among human immunodeficiency virus-positive subjects: epidemiology and outcome after two decades of HAART era. World J Virol 2015; 4:209. 26279983 10.5501/wjv.v4.i3.209 PMC4534813

[28] Palella FJ Jr, Baker RK, Moorman AC, et al Mortality in the highly active antiretroviral therapy era: changing causes of death and disease in the HIV outpatient study. J Acquir Immune Defic Syndr 2006; 43:27–34. 16878047 10.1097/01.qai.0000233310.90484.16

[29] Eyawo O, Franco-Villalobos C, Hull MW, et al Changes in mortality rates and causes of death in a population-based cohort of persons living with and without HIV from 1996 to 2012. BMC Infect Dis 2017; 17:174. 28241797 10.1186/s12879-017-2254-7 PMC5329918

[30] Ministerio de Sanidad . Gobierno de España. Strategic plan for the prevention and control of HIV and other sexually transmitted infections in Spain, 2021–2030. Madrid: 2023. Available at: https://www.sanidad.gob.es/ciudadanos/enfLesiones/enfTransmisibles/sida/planNalSida/PLAN_FOR_THE_PREVENTION_AND_CONTROL_OF_HIV_AND_OTHER_SEXUALLY_TRANSMITTED_INFECTIONS_IN_SPAIN.pdf. Accessed 01 July 2023.

[31] Weisberg DF, Gordon KS, Barry DT, et al Long-term prescription opioids and/or benzodiazepines and mortality among HIV-infected and uninfected patients. J Acquir Immune Defic Syndr 2015; 69:223–33. 26009831 10.1097/QAI.0000000000000591 PMC4446730

[32] Lee C-Y, Lin Y-P, Tu H-P, Wang S-F, Lu P-L. Sex stratification of the trends and risk of mortality among individuals living with HIV under different transmission categories. Sci Rep 2022; 12:9266. 35661129 10.1038/s41598-022-13294-y PMC9166722

[33] Mascolini M. Trends in mortality in people living with HIV in an international cohort (RESPOND). 30th CROI, Conf retroviruses opportunistic infect 2023; Abstract 870. Available at: https://www.natap.org/2023/CROI/croi_08.htm. Accessed 15 October 2023.

