# Supplementary material for: Evolving AIDS- and non-AIDS Mortality and Predictors in the PISCIS Cohort of People Living With HIV in Catalonia and the Balearic Islands (Spain), 1998–2020
Source: Open Forum Infect Dis. 2024 Mar 8;11(4):ofae132. doi: 10.1093/ofid/ofae132 (PMC10977910; doi:10.1093/ofid/ofae132)
Supplement: ofae132_Supplementary_Data [file ofae132_supplementary_data.docx]

**Supplementary materials**

**Tables**

**Table S1: Median age and per calendar period and cause of death**

| **Causes of death** | **1998 - 2003** | **2004 - 2008** | **2009 - 2014** | **2015 - 2020** | **p-value** |
| --- | --- | --- | --- | --- | --- |
| **Accidents** | 38.93 (35.28; 43.82) | 37.06 (34.6; 45.49) | 44.53 (39.25; 48.26) | 51.24 (41.42; 56.33) | 0.001 |
| **Chronic respiratory** | 38.09 (33.05; 52.99) | 51.01 (38.72; 67.12) | 48.13 (44.04; 53.09) | 57.61 (49.55; 63.83) | < 0.001 |
| **COVID-19** | - | - | - | 61.55 (54.01; 66.35) | - |
| **CVD** | 44.12 (37.54; 53.6) | 45.43 (39.42; 55.39) | 48.73 (44.01; 54.71) | 55.01 (49.51; 67) | < 0.001 |
| **Gastrointestinal** | 41.02 (37.39; 43.2) | 40.65 (36.18; 66.12) | 47.68 (44.81; 55.08) | 56.56 (47.44; 64.28) | 0.006 |
| **Hepatitis** | 40.52 (36.95; 45.13) | 43.53 (39.85; 47.22) | 48.44 (44.64; 52.6) | 53.45 (49.78; 57.62) | < 0.001 |
| **Liver disease** | 41.22 (35.48; 45.74) | 42.59 (37.97; 47.55) | 48.89 (44.76; 52.36) | 52.31 (48.78; 56.89) | < 0.001 |
| **Neuropsychiatric** | 41.94 (38.82; 48.92) | 44.45 (40.35; 58.3) | 48.6 (44.3; 56.98) | 50.45 (43.31; 57.38) | 0.224 |
| **Non-AIDS cancers** | 43.22 (36.65; 51.68) | 48.13 (41.72; 57.93) | 51.74 (46.66; 58.27) | 56.2 (51.96; 62.72) | < 0.001 |
| **Renal disease** | 38.38 (35.84; 50.26) | 46.4 (38.51; 54.42) | 44.52 (39.91; 62.93) | 54.19 (50.35; 57.56) | 0.164 |
| **Substance abuse** | 33.27 (30.93; 36.68) | 36.55 (32.85; 42.52) | 41.99 (37.59; 47.41) | 47.7 (44.94; 50.85) | < 0.001 |
| **Suicide** | 41.24 (38.05; 42.27) | 39.66 (36.58; 41.2) | 44.16 (38.71; 48.88) | 49.03 (42.91; 53.87) | 0.013 |
| **Others** | 41.98 (34.55; 45.05) | 42.3 (36.82; 47.29) | 46.22 (40.81; 54.17) | 53.15 (48.49; 60.06) | < 0.001 |
| **Unknown** | 37.19 (34.19; 42.69) | 39.22 (35.59; 44.41) | 44.74 (39.83; 50.44) | 52.99 (46; 59.61) | < 0.001 |

Abbreviations: CVD, cardiovascular disease; COVID-19, coronavirus disease 2019

**Table S2: Cohort characteristics and crude mortality rates of different sociodemographic and clinical subgroups with missing variables imputed.**

|  | **Overall cohort N (%)** | **Dead N (%)** | **p-value** | **PY (x1000)** | **CMR per 1000 PY (95% CI)** |
| --- | --- | --- | --- | --- | --- |
| **Age at cohort entry (years)** |  |  | <0.0001 |  |  |
| 16-29 | 8436 (28.12) | 552 (14.01) |  | 88133.89 | 6.26 (6.25; 6.28) |
| 30-39 | 12155 (40.52) | 1528 (38.79) |  | 128647.83 | 11.88 (11.86; 11.9) |
| 40-49 | 6455 (21.52) | 1078 (27.37) |  | 62095.11 | 17.36 (17.33; 17.39) |
| 50-64 | 2561 (8.54) | 575 (14.6) |  | 22442.29 | 25.62 (25.56; 25.69) |
| ≥65 | 389 (1.3) | 206 (5.23) |  | 2736.64 | 75.27 (74.95; 75.6) |
| **Age at cohort entry, median (IQR), years** | 35.16 (29.25; 42.02) | 39.32 (33.17; 47.68) | <0.0001 |  |  |
| **Age at death or last contact, median (IQR), years** | 46.09 (37.87; 54.46) | 48.04 (41.09; 56.03) | <0.0001 |  |  |
| **Sex** |  |  | 0.006 |  |  |
| Male | 24541 (81.81) | 3160 (80.22) |  | 238271.67 | 13.26 (13.25; 13.28) |
| Female | 5455 (18.19) | 779 (19.78) |  | 65784.1 | 11.84 (11.82; 11.87) |
| **Region of origin** |  |  | <0.0001 |  |  |
| Spanish | 18556 (61.1) | 3577 (87.2) |  | 203889.1 | 17.54 (17.53-17.56) |
| Non-Spanish | 11838 (39.0) | 525 (12.8) |  | 81477.8 | 6.44 (6.43-6.46) |
| **Socioeconomic status** |  |  | <0.0001 |  |  |
| Least socioeconomic deprivation | 15185 (50.62) | 1539 (39.07) |  | 142249.94 | 10.82 (10.8; 10.84) |
| Mild socioeconomic deprivation | 5827 (19.43) | 919 (23.33) |  | 63894.24 | 14.38 (14.35; 14.41) |
| Moderate/severe socioeconomic deprivation | 8984 (29.95) | 1481 (37.6) |  | 97911.59 | 15.13 (15.1; 15.15) |
| **HIV transmission route** |  |  | <0.0001 |  |  |
| MSM | 15109 (50.37) | 715 (18.15) |  | 134010.69 | 5.34 (5.32; 5.35) |
| PWID | 5461 (18.21) | 1953 (49.58) |  | 68601.04 | 28.47 (28.43; 28.51) |
| Male heterosexual | 4428 (14.76) | 777 (19.73) |  | 44952.95 | 17.28 (17.25; 17.32) |
| Women infected through sex | 3995 (13.32) | 351 (8.91) |  | 47273.31 | 7.42 (7.4; 7.45) |
| Other | 1003 (3.34) | 143 (3.63) |  | 9217.77 | 15.51 (15.43; 15.59) |
| **Period of HIV diagnosis** |  |  | <0.0001 |  |  |
| 1981-1997 | 4953 (16.51) | 1569 (39.83) |  | 72251.5 | 21.72 (21.68; 21.75) |
| 1998-2003 | 5943 (19.81) | 1375 (34.91) |  | 87305.81 | 15.75 (15.72; 15.78) |
| 2004-2008 | 5708 (19.03) | 605 (15.36) |  | 65561.42 | 9.23 (9.2; 9.25) |
| 2009-2014 | 7078 (23.6) | 295 (7.49) |  | 57256.68 | 5.15 (5.13; 5.17) |
| 2015-2020 | 5712 (18.8) | 74 (1.8) |  | 14792.8 | 5 (5.0-5.0) |
| **Years since HIV diagnosis, median (IQR)** | 11.45 (5.73; 18.37) | 11.62 (4.96; 18.78) | <0.0001 |  |  |
| **CD4 count at cohort entry, cells/µL** |  |  | <0.0001 |  |  |
| <200 | 6238 (20.8) | 1343 (34.09) |  | 64887.07 | 20.7 (20.66; 20.73) |
| 200-349 | 4731 (15.77) | 536 (13.61) |  | 46818.44 | 11.45 (11.42; 11.48) |
| 350-499 | 6780 (22.6) | 763 (19.37) |  | 69854.25 | 10.92 (10.9; 10.95) |
| ≥500 | 12247 (40.83) | 1297 (32.93) |  | 122496 | 10.59 (10.57; 10.61) |
| **CD4 count (cells/µL), median (IQR)** | 434 (240; 640) | 364 (131; 594) | <0.0001 |  |  |
| **HIV-RNA at cohort entry** |  |  | <0.0001 |  |  |
| Detectable | 3488 (11.63) | 313 (7.95) |  | 24441.81 | 12.81 (12.76; 12.85) |
| Undetectable | 26508 (88.37) | 3626 (92.05) |  | 279613.96 | 12.97 (12.95; 12.98) |
| **AIDS-defining illness ever?** |  |  | <0.0001 |  |  |
| No | 24692 (82.32) | 2386 (60.57) |  | 240730.18 | 9.91 (9.9; 9.92) |
| Yes | 5304 (17.68) | 1553 (39.43) |  | 63325.59 | 24.52 (24.49; 24.56) |
| **ART at death or last contact** |  |  | <0.0001 |  |  |
| Yes | 26692 (88.99) | 2784 (70.68) |  | 285590.33 | 9.75 (9.74; 9.76) |
| No | 3304 (11.01) | 1155 (29.32) |  | 18465.44 | 62.55 (62.44; 62.66) |
| **Years on ART, median (IQR)** | 7.58 (3; 13.21) | 5.16 (1.22; 10.72) | <0.0001 |  |  |
| **Comorbidities** |  |  |  |  |  |
| Myocardial infarction | 683 (2.28) | 164 (4.16) | <0.0001 | 9964.39 | 16.46 (16.38; 16.54) |
| Congestive heart failure | 678 (2.26) | 254 (6.45) | <0.0001 | 9558.86 | 26.57 (26.47; 26.68) |
| Peripheral vascular disease | 488 (1.63) | 146 (3.71) | <0.0001 | 7238.25 | 20.17 (20.07; 20.27) |
| Cerebrovascular disease | 1231 (4.1) | 363 (9.22) | <0.0001 | 15865.04 | 22.88 (22.81; 22.95) |
| Dementia | 165 (0.55) | 79 (2.01) | <0.0001 | 2096.13 | 37.69 (37.43; 37.95) |
| Chronic pulmonary disease | 4999 (16.67) | 895 (22.72) | <0.0001 | 64844.56 | 13.8 (13.77; 13.83) |
| Rheumatoid disease | 168 (0.56) | 20 (0.51) | 0.763 | 2191.41 | 9.13 (9; 9.25) |
| Peptic ulcer disease | 427 (1.42) | 98 (2.49) | <0.0001 | 5534.98 | 17.71 (17.59; 17.82) |
| Mild liver disease | 1209 (4.03) | 602 (15.28) | <0.0001 | 16150.11 | 37.28 (37.18; 37.37) |
| Diabetes without chronic complications | 1802 (6.01) | 402 (10.21) | <0.0001 | 24856.12 | 16.17 (16.12; 16.22) |
| Diabetes with chronic complications | 299 (1) | 93 (2.36) | <0.0001 | 4155.69 | 22.38 (22.24; 22.52) |
| Hemiplegia or paraplegia | 369 (1.23) | 121 (3.07) | <0.0001 | 4474.57 | 27.04 (26.89; 27.19) |
| Renal disease | 1148 (3.83) | 338 (8.58) | <0.0001 | 16061.42 | 21.04 (20.97; 21.12) |
| Cancer (any malignancy) | 3166 (10.55) | 1234 (31.33) | <0.0001 | 36838.55 | 33.5 (33.44; 33.56) |
| Moderate or severe liver disease | 626 (2.09) | 356 (9.04) | <0.0001 | 8247.75 | 43.16 (43.02; 43.31) |
| Metastatic solid tumour | 683 (2.28) | 492 (12.49) | <0.0001 | 7780.3 | 63.24 (63.06; 63.41) |
| Number of comorbidities, median (IQR) | 0 (0; 1) | 1 (0; 2) | <0.0001 |  |  |
| **Number of comorbidities** |  |  | <0.0001 |  |  |
| 0 | 19450 (64.84) | 1449 (36.79) |  | 173774.91 | 8.34 (8.32; 8.35) |
| 1 | 6330 (21.1) | 955 (24.24) |  | 73327.6 | 13.02 (13; 13.05) |
| 2 | 2329 (7.76) | 676 (17.16) |  | 30152.73 | 22.42 (22.37; 22.47) |
| ≥3 | 1887 (6.29) | 859 (21.81) |  | 26800.52 | 32.05 (31.98; 32.12) |

Abbreviations: PY, person-years; CMR, crude mortality rate; IQR, interquartile range; CI, confidence interval; PWID, people who inject drugs; MSM, men who have sex with men; ART, antiretroviral therapy, undetectable HIV-RNA was defined as ≤50 copies/ml.

**Table S3: Descriptive characteristics of deaths in the cohort of PLWH according to calendar periods.**

|  | **1998-2003** | **2004-2008** | **2009-2014** | **2015-2020** | **p-value** |
| --- | --- | --- | --- | --- | --- |
| **Age at cohort entry (years)** |  |  |  |  | <0.0001 |
| 16-29 | 99 (15.11) | 158 (15.93) | 183 (14.51) | 174 (14.57) |  |
| 30-39 | 286 (43.66) | 427 (43.04) | 499 (39.57) | 419 (35.09) |  |
| 40-49 | 156 (23.82) | 236 (23.79) | 353 (27.99) | 344 (28.81) |  |
| 50-64 | 75 (11.45) | 118 (11.9) | 173 (13.72) | 198 (16.58) |  |
| ≥65 | 39 (5.95) | 53 (5.34) | 53 (4.2) | 59 (4.94) |  |
| **Age at cohort entry, median (IQR), years** | 38.05 (32.98; 45.83) | 38.15 (32.32; 45.91) | 39.05 (33.07; 46.29) | 40.17 (33.12; 48.49) | <0.0001 |
| **Age at death or last contact, median (IQR), years** | 40.48 (35.23; 47.41) | 43.19 (37.95; 50.42) | 47.59 (42.27; 53.45) | 53.42 (47.76; 59.93) | <0.0001 |
| **Sex** |  |  |  |  | 0.097 |
| Male | 529 (80.76) | 814 (82.06) | 1000 (79.3) | 929 (77.81) |  |
| Female | 126 (19.24) | 178 (17.94) | 261 (20.7) | 264 (22.11) |  |
| Missing | 0 (0) | 0 (0) | 0 (0) | 1 (0.08) |  |
| **Region of origin** |  |  |  |  | <0.0001 |
| Spanish | 525 (80.15) | 837 (84.38) | 1040 (82.47) | 1002 (83.92) |  |
| Not Spanish | 46 (7.02) | 96 (9.68) | 173 (13.72) | 181 (15.16) |  |
| Missing | 84 (12.82) | 59 (5.95) | 48 (3.81) | 11 (0.92) |  |
| **Socioeconomic status** |  |  |  |  | 0.67 |
| Least socioeconomic deprivation | 180 (27.48) | 271 (27.32) | 389 (30.85) | 399 (33.42) |  |
| Mild socioeconomic deprivation | 106 (16.18) | 186 (18.75) | 239 (18.95) | 227 (19.01) |  |
| Moderate/severe socioeconomic deprivation | 173 (26.41) | 304 (30.65) | 389 (30.85) | 429 (35.93) |  |
| Missing | 196 (29.92) | 231 (23.29) | 244 (19.35) | 139 (11.64) |  |
| **HIV transmission route** |  |  |  |  | <0.0001 |
| MSM | 80 (12.21) | 142 (14.31) | 180 (14.27) | 208 (17.42) |  |
| PWID | 337 (51.45) | 503 (50.71) | 634 (50.28) | 528 (44.22) |  |
| Male heterosexual | 112 (17.1) | 174 (17.54) | 226 (17.92) | 231 (19.35) |  |
| Women infected through sex | 47 (7.18) | 68 (6.85) | 102 (8.09) | 127 (10.64) |  |
| Other | 25 (3.82) | 44 (4.44) | 25 (1.98) | 36 (3.02) |  |
| Missing | 54 (8.24) | 61 (6.15) | 94 (7.45) | 64 (5.36) |  |
| **Period of HIV diagnosis** |  |  |  |  | <0.0001 |
| 1981-1997 | 294 (44.89) | 421 (42.44) | 504 (39.97) | 434 (36.35) |  |
| 1998-2003 | 361 (55.11) | 377 (38) | 372 (29.5) | 332 (27.81) |  |
| 2004-2008 | 0 (0) | 194 (19.56) | 235 (18.64) | 204 (17.09) |  |
| 2009-2014 | 0 (0) | 0 (0) | 150 (11.9) | 150 (12.56) |  |
| 2015-2021 | 0 (0) | 0 (0) | 0 (0) | 74 (6.2) |  |
| **Years since HIV diagnosis, median (IQR)** | 3.44 (0.84; 9.61) | 7.8 (3.51; 13.99) | 12.08 (6.8; 19.19) | 17.71 (11.12; 24.29) | <0.0001 |
| **CD4 count at cohort entry, cells/µL** |  |  |  |  | <0.0001 |
| <200 | 275 (41.98) | 315 (31.75) | 333 (26.41) | 272 (22.78) |  |
| 200-349 | 71 (10.84) | 143 (14.42) | 172 (13.64) | 164 (13.74) |  |
| 350-499 | 42 (6.41) | 80 (8.06) | 123 (9.75) | 116 (9.72) |  |
| ≥500 | 93 (14.2) | 150 (15.12) | 220 (17.45) | 200 (16.75) |  |
| Missing | 174 (26.56) | 304 (30.65) | 413 (32.75) | 442 (37.02) |  |
| **CD4 count (cells/µL), median (IQR)** | 155 (51; 378) | 230 (80; 448) | 264.5 (112.75; 506.25) | 280 (127.75; 520) | <0.0001 |
| **HIV-RNA at cohort entry** |  |  |  |  | <0.0001 |
| Detectable | 4 (0.61) | 14 (1.41) | 42 (3.33) | 31 (2.6) |  |
| Undetectable | 433 (66.11) | 648 (65.32) | 781 (61.93) | 712 (59.63) |  |
| Missing | 218 (33.28) | 330 (33.27) | 438 (34.73) | 451 (37.77) |  |
| **History of AIDS-defining illness** |  |  |  |  | <0.0001 |
| No | 341 (52.06) | 587 (59.17) | 800 (63.44) | 769 (64.41) |  |
| Yes | 314 (47.94) | 405 (40.83) | 461 (36.56) | 425 (35.59) |  |
| **ART at death or last contact** |  |  |  |  | <0.0001 |
| Yes | 280 (42.75) | 515 (51.92) | 952 (75.5) | 1087 (91.04) |  |
| No | 375 (57.25) | 477 (48.08) | 309 (24.5) | 107 (8.96) |  |
| **Years on ART, median (IQR)** | 1.42 (0.38; 2.89) | 3.96 (1.28; 6.58) | 6.15 (2.56; 10.42) | 11.06 (6.59; 16.46) | <0.0001 |
| **Comorbidities** |  |  |  |  |  |
| Myocardial infarction | 0 (0) | 7 (0.71) | 38 (3.01) | 74 (6.2) | <0.0001 |
| Congestive heart failure | 0 (0) | 9 (0.91) | 66 (5.23) | 130 (10.89) | <0.0001 |
| Peripheral vascular disease | 0 (0) | 3 (0.3) | 33 (2.62) | 115 (9.63) | <0.0001 |
| Cerebrovascular disease | 0 (0) | 15 (1.51) | 67 (5.31) | 155 (12.98) | <0.0001 |
| Dementia | 0 (0) | 7 (0.71) | 26 (2.06) | 47 (3.94) | 0.009 |
| Chronic pulmonary disease | 1 (0.15) | 33 (3.33) | 237 (18.79) | 436 (36.52) | <0.0001 |
| Rheumatoid disease | 0 (0) | 1 (0.1) | 4 (0.32) | 13 (1.09) | 0.086 |
| Peptic ulcer disease | 0 (0) | 3 (0.3) | 13 (1.03) | 46 (3.85) | <0.0001 |
| Mild liver disease | 5 (0.76) | 96 (9.68) | 540 (42.82) | 683 (57.2) | <0.0001 |
| Diabetes without chronic complications | 1 (0.15) | 17 (1.71) | 101 (8.01) | 172 (14.41) | <0.0001 |
| Diabetes with chronic complications | 0 (0) | 1 (0.1) | 21 (1.67) | 55 (4.61) | <0.0001 |
| Hemiplegia or paraplegia | 0 (0) | 2 (0.2) | 44 (3.49) | 61 (5.11) | <0.0001 |
| Renal disease | 1 (0.15) | 9 (0.91) | 87 (6.9) | 171 (14.32) | <0.0001 |
| Cancer (any malignancy) | 12 (1.83) | 78 (7.86) | 324 (25.69) | 430 (36.01) | <0.0001 |
| Moderate or severe liver disease | 0 (0) | 3 (0.3) | 136 (10.79) | 154 (12.9) | <0.0001 |
| Metastatic solid tumour | 0 (0) | 5 (0.5) | 116 (9.2) | 218 (18.26) | <0.0001 |
| **Number of comorbidities, median (IQR)** | 0 (0; 1) | 0 (0; 1) | 2 (1; 3) | 2 (1; 4) | <0.0001 |
| **Number of comorbidities** |  |  |  |  | <0.0001 |
| 0 | 32 (62.75) | 250 (54.35) | 230 (21.93) | 122 (10.87) |  |
| 1 | 18 (35.29) | 147 (31.96) | 285 (27.17) | 207 (18.45) |  |
| 2 | 1 (1.96) | 48 (10.43) | 237 (22.59) | 244 (21.75) |  |
| ≥3 | 0 (0) | 15 (3.26) | 297 (28.31) | 549 (48.93) |  |

Abbreviations: PY, person-years; CMR, crude mortality rate; IQR, interquartile range; CI, confidence interval; PWID, people who inject drugs; MSM, men who have sex with men; ART, antiretroviral therapy, undetectable HIV-RNA was defined as ≤50 copies/ml.

**Table S4: Proportions of mortality causes by calendar periods**

|  | **1998-2003** | **2004-2008** | **2009-2014** | **2015-2020** | **p-value** |
| --- | --- | --- | --- | --- | --- |
| **AIDS** | 252 (38.5) | 257 (25.9) | 284 (22.5) | 115 (9.8) | < 0.0001 |
| **Infection** | 19 (2.9) | 52 (5.2) | 46 (3.7) | 62 (5.9) | 0.118 |
| **Hepatitis** | 31 (4.7) | 55 (5.5) | 72 (5.7) | 54 (4.6) | 0.763 |
| **CVD** | 40 (6.1) | 69 (7.0) | 111 (8.8) | 159 (13.5) | < 0.0001 |
| **AIDS-defining cancers** | 52 (7.9) | 70 (7.1) | 68 (5.4) | 40 (3.4) | < 0.0001 |
| **Non-AIDS cancers** | 53 (8.1) | 139 (14.0) | 238 (18.9) | 260 (22.1) | < 0.0001 |
| **Liver disease** | 41 (6.3) | 70 (7.1) | 61 (4.8) | 42 (3.6) | 0.0001 |
| **Renal disease** | 12 (1.8) | 21 (2.1) | 13 (1.0) | 13 (1.1) | 0.049 |
| **Accidents** | 16 (2.4) | 28 (2.8) | 47 (3.7) | 35 (3.0) | 0.398 |
| **Suicide** | 9 (1.4) | 12 (1.2) | 34 (2.7) | 21 (1.8) | 0.185 |
| **Substance abuse** | 40 (6.1) | 41 (4.1) | 55 (4.4) | 55 (4.7) | 0.347 |
| **Chronic respiratory** | 11 (1.7) | 25 (2.5) | 46 (3.7) | 46 (3.9) | 0.003 |
| **Gastrointestinal** | 10 (1.5) | 18 (1.8) | 17 (1.4) | 12 (1.0) | 0.191 |
| **Neuropsychiatric** | 15 (2.3) | 22 (2.2) | 24 (1.9) | 35 (3.0) | 0.349 |
| **Others** | 26 (4.0) | 69 (7.0) | 66 (5.2) | 55 (4.7) | 0.67 |
| **Unknown** | 28 (4.3) | 44 (4.4) | 79 (6.3) | 171 (14.6) | < 0.0001 |
| **Total** | 655 (100.0) | 992 (100.0) | 1261 (100.0) | 1175 (100.0) |  |

Abbreviations: CVD, cardiovascular disease.

**Table S5: Proportions of mortality causes by years**

|  | **1998** | **1999** | **2000** | **2001** | **2002** | **2003** | **2004** | **2005** | **2006** | **2007** | **2008** | **2009** |
| --- | --- | --- | --- | --- | --- | --- | --- | --- | --- | --- | --- | --- |
| **AIDS** | 18 (47.37) | 26 (48.15) | 47 (40.87) | 37 (29.37) | 50 (39.06) | 74 (38.14) | 51 (32.08) | 36 (20.34) | 50 (22.42) | 65 (30.66) | 55 (24.89) | 43 (19.28) |
| **Infection** | 0 (0) | 3 (5.56) | 6 (5.22) | 3 (2.38) | 2 (1.56) | 5 (2.58) | 4 (2.52) | 14 (7.91) | 13 (5.83) | 8 (3.77) | 13 (5.88) | 9 (4.04) |
| **Hepatitis** | 0 (0) | 2 (3.7) | 7 (6.09) | 5 (3.97) | 6 (4.69) | 11 (5.67) | 9 (5.66) | 10 (5.65) | 15 (6.73) | 10 (4.72) | 11 (4.98) | 19 (8.52) |
| **CVD** | 3 (7.89) | 2 (3.7) | 7 (6.09) | 12 (9.52) | 9 (7.03) | 7 (3.61) | 9 (5.66) | 12 (6.78) | 15 (6.73) | 16 (7.55) | 17 (7.69) | 15 (6.73) |
| **AIDS-defining cancers** | 0 (0) | 5 (9.26) | 8 (6.96) | 14 (11.11) | 9 (7.03) | 16 (8.25) | 9 (5.66) | 15 (8.47) | 15 (6.73) | 13 (6.13) | 18 (8.14) | 12 (5.38) |
| **Non-AIDS cancers** | 5 (13.16) | 1 (1.85) | 6 (5.22) | 12 (9.52) | 11 (8.59) | 18 (9.28) | 23 (14.47) | 19 (10.73) | 36 (16.14) | 30 (14.15) | 31 (14.03) | 38 (17.04) |
| **Liver disease** | 0 (0) | 6 (11.11) | 8 (6.96) | 8 (6.35) | 9 (7.03) | 10 (5.15) | 8 (5.03) | 19 (10.73) | 17 (7.62) | 15 (7.08) | 11 (4.98) | 11 (4.93) |
| **Renal disease** | 0 (0) | 0 (0) | 1 (0.87) | 3 (2.38) | 4 (3.12) | 4 (2.06) | 4 (2.52) | 4 (2.26) | 3 (1.35) | 3 (1.42) | 7 (3.17) | 5 (2.24) |
| **Accidents** | 1 (2.63) | 0 (0) | 5 (4.35) | 3 (2.38) | 2 (1.56) | 5 (2.58) | 3 (1.89) | 7 (3.95) | 10 (4.48) | 3 (1.42) | 5 (2.26) | 8 (3.59) |
| **Suicide** | 0 (0) | 1 (1.85) | 1 (0.87) | 2 (1.59) | 2 (1.56) | 3 (1.55) | 4 (2.52) | 1 (0.56) | 2 (0.9) | 3 (1.42) | 2 (0.9) | 3 (1.35) |
| **Substance abuse** | 2 (5.26) | 5 (9.26) | 6 (5.22) | 15 (11.9) | 6 (4.69) | 6 (3.09) | 11 (6.92) | 10 (5.65) | 6 (2.69) | 8 (3.77) | 6 (2.71) | 10 (4.48) |
| **Chronic respiratory** | 2 (5.26) | 0 (0) | 1 (0.87) | 0 (0) | 2 (1.56) | 6 (3.09) | 2 (1.26) | 4 (2.26) | 8 (3.59) | 6 (2.83) | 5 (2.26) | 12 (5.38) |
| **Gastrointestinal** | 0 (0) | 0 (0) | 1 (0.87) | 2 (1.59) | 1 (0.78) | 6 (3.09) | 3 (1.89) | 3 (1.69) | 2 (0.9) | 3 (1.42) | 7 (3.17) | 2 (0.9) |
| **Neuropsychiatric** | 1 (2.63) | 1 (1.85) | 2 (1.74) | 3 (2.38) | 4 (3.12) | 4 (2.06) | 3 (1.89) | 5 (2.82) | 7 (3.14) | 1 (0.47) | 6 (2.71) | 5 (2.24) |
| **Others** | 5 (13.16) | 0 (0) | 3 (2.61) | 2 (1.59) | 7 (5.47) | 9 (4.64) | 13 (8.18) | 9 (5.08) | 13 (5.83) | 15 (7.08) | 19 (8.6) | 17 (7.62) |
| **Unknown** | 1 (2.63) | 2 (3.7) | 6 (5.22) | 5 (3.97) | 4 (3.12) | 10 (5.15) | 3 (1.89) | 9 (5.08) | 11 (4.93) | 13 (6.13) | 8 (3.62) | 14 (6.28) |
| **Total** | 38 (100) | 54 (100) | 115 (100) | 126 (100) | 128 (100) | 194 (100) | 159 (100) | 177 (100) | 223 (100) | 212 (100) | 221 (100) | 223 (100) |
|  | **2010** | **2011** | **2012** | **2013** | **2014** | **2015** | **2016** | **2017** | **2018** | **2019** | **2020** | **p-value** |
| **AIDS** | 61 (28.91) | 54 (25.12) | 39 (20.21) | 46 (21.9) | 41 (19.62) | 39 (19.5) | 24 (12.9) | 13 (7.26) | 16 (8.33) | 12 (5.97) | 11 (5.07) | < 0.001 |
| **Infection** | 7 (3.32) | 13 (6.05) | 9 (4.66) | 5 (2.38) | 3 (1.44) | 8 (4) | 18 (9.68) | 10 (5.59) | 5 (2.6) | 11 (5.47) | 10 (4.61) | 0.268 |
| **Hepatitis** | 14 (6.64) | 12 (5.58) | 10 (5.18) | 5 (2.38) | 12 (5.74) | 16 (8) | 12 (6.45) | 5 (2.79) | 14 (7.29) | 4 (1.99) | 3 (1.38) | 0.239 |
| **CVD** | 20 (9.48) | 18 (8.37) | 15 (7.77) | 17 (8.1) | 26 (12.44) | 24 (12) | 25 (13.44) | 31 (17.32) | 35 (18.23) | 32 (15.92) | 12 (5.53) | < 0.001 |
| **AIDS-defining cancers** | 10 (4.74) | 15 (6.98) | 8 (4.15) | 13 (6.19) | 10 (4.78) | 8 (4) | 10 (5.38) | 6 (3.35) | 7 (3.65) | 7 (3.48) | 2 (0.92) | < 0.001 |
| **Non-AIDS cancers** | 33 (15.64) | 33 (15.35) | 42 (21.76) | 43 (20.48) | 49 (23.44) | 41 (20.5) | 38 (20.43) | 50 (27.93) | 48 (25) | 60 (29.85) | 23 (10.6) | < 0.001 |
| **Liver disease** | 5 (2.37) | 19 (8.84) | 7 (3.63) | 10 (4.76) | 9 (4.31) | 4 (2) | 10 (5.38) | 6 (3.35) | 3 (1.56) | 13 (6.47) | 6 (2.76) | 0.001 |
| **Renal disease** | 0 (0) | 3 (1.4) | 0 (0) | 4 (1.9) | 1 (0.48) | 3 (1.5) | 3 (1.61) | 3 (1.68) | 3 (1.56) | 1 (0.5) | 0 (0) | 0.042 |
| **Accidents** | 8 (3.79) | 7 (3.26) | 12 (6.22) | 9 (4.29) | 3 (1.44) | 3 (1.5) | 3 (1.61) | 6 (3.35) | 11 (5.73) | 10 (4.98) | 2 (0.92) | 0.486 |
| **Suicide** | 5 (2.37) | 6 (2.79) | 8 (4.15) | 4 (1.9) | 8 (3.83) | 6 (3) | 2 (1.08) | 3 (1.68) | 5 (2.6) | 3 (1.49) | 2 (0.92) | 0.209 |
| **Substance abuse** | 8 (3.79) | 9 (4.19) | 7 (3.63) | 12 (5.71) | 9 (4.31) | 11 (5.5) | 10 (5.38) | 10 (5.59) | 8 (4.17) | 13 (6.47) | 3 (1.38) | 0.151 |
| **Chronic respiratory** | 4 (1.9) | 5 (2.33) | 9 (4.66) | 9 (4.29) | 7 (3.35) | 5 (2.5) | 5 (2.69) | 12 (6.7) | 10 (5.21) | 10 (4.98) | 4 (1.84) | 0.004 |
| **Gastrointestinal** | 1 (0.47) | 1 (0.47) | 2 (1.04) | 5 (2.38) | 6 (2.87) | 2 (1) | 2 (1.08) | 2 (1.12) | 2 (1.04) | 2 (1) | 2 (0.92) | 0.548 |
| **Neuropsychiatric** | 4 (1.9) | 5 (2.33) | 3 (1.55) | 4 (1.9) | 3 (1.44) | 3 (1.5) | 9 (4.84) | 1 (0.56) | 6 (3.12) | 7 (3.48) | 9 (4.15) | 0.277 |
| **Others** | 16 (7.58) | 9 (4.19) | 12 (6.22) | 9 (4.29) | 3 (1.44) | 12 (6) | 8 (4.3) | 10 (5.59) | 8 (4.17) | 8 (3.98) | 9 (4.15) | 0.293 |
| **Unknown** | 15 (7.11) | 6 (2.79) | 10 (5.18) | 15 (7.14) | 19 (9.09) | 15 (7.5) | 7 (3.76) | 11 (6.15) | 11 (5.73) | 8 (3.98) | 119 (54.84) | < 0.001 |
| **Total** | 211 (100) | 215 (100) | 193 (100) | 210 (100) | 209 (100) | 200 (100) | 186 (100) | 179 (100) | 192 (100) | 201 (100) | 217 (100) |  |

Abbreviations: CVD, cardiovascular disease.

**Figures**

**Figure S1: Distribution of AIDS-related and non-AIDS-related cancers mortality among PLWH**


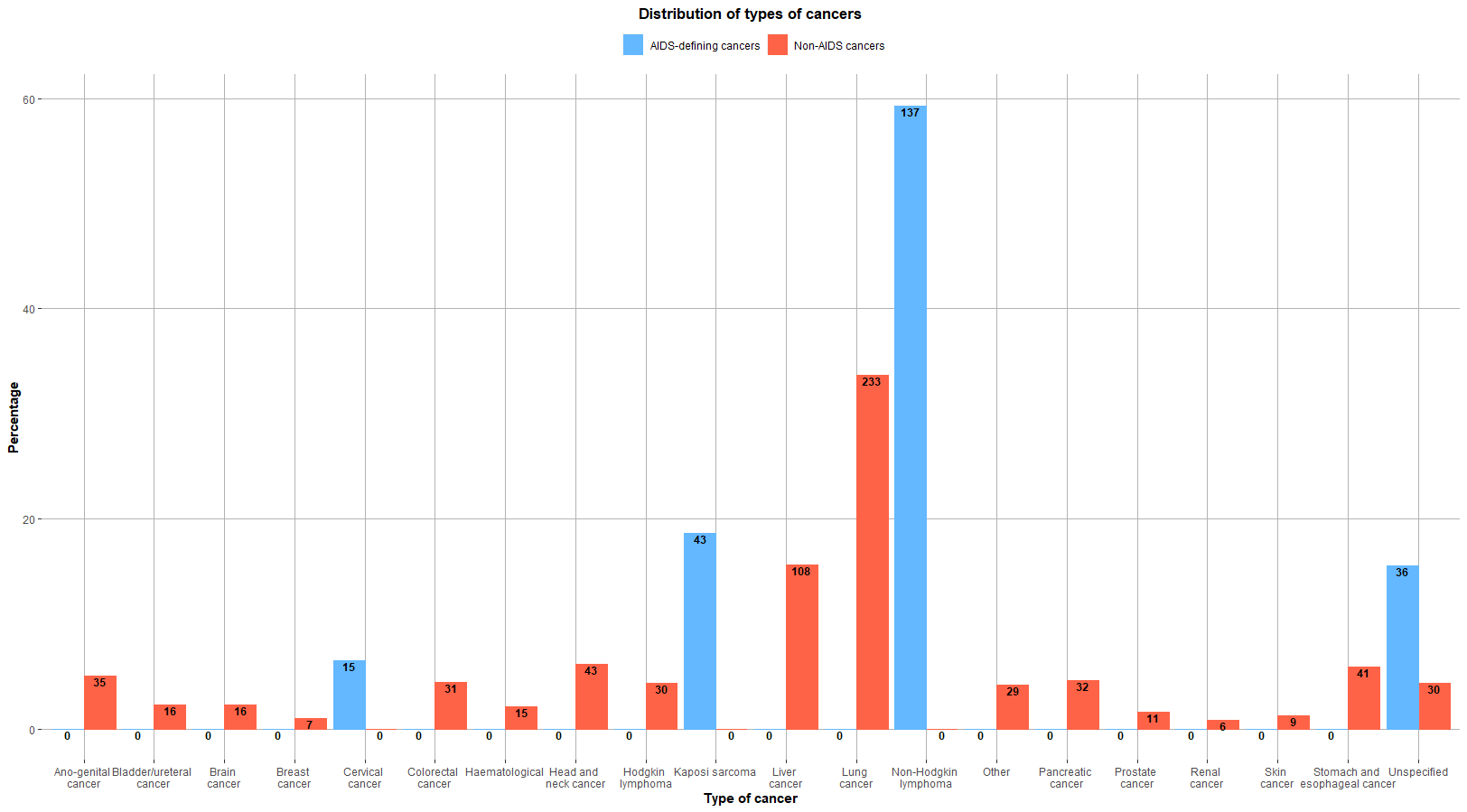


**Figure S2: Causes of death among people living with HIV in Catalonia and the Balearic Islands, Spain, 1998 – 2020, by years**


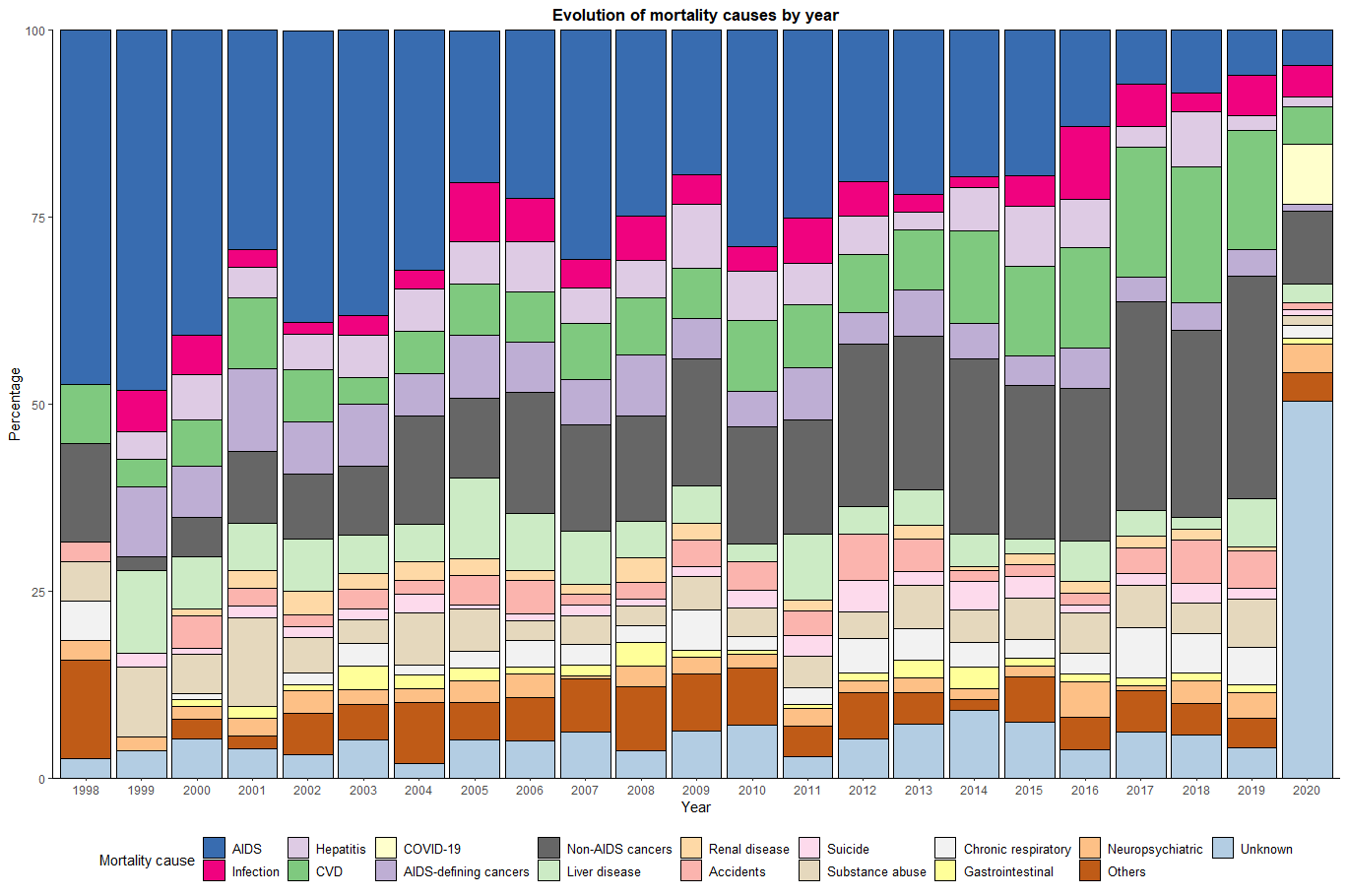


Abbreviations: CVD, cardiovascular disease; COVID-19, coronavirus disease 2019

**Figure S3: Causes of death among people living with HIV in Catalonia and the Balearic Islands, Spain, by years since of enrolment into cohort.**


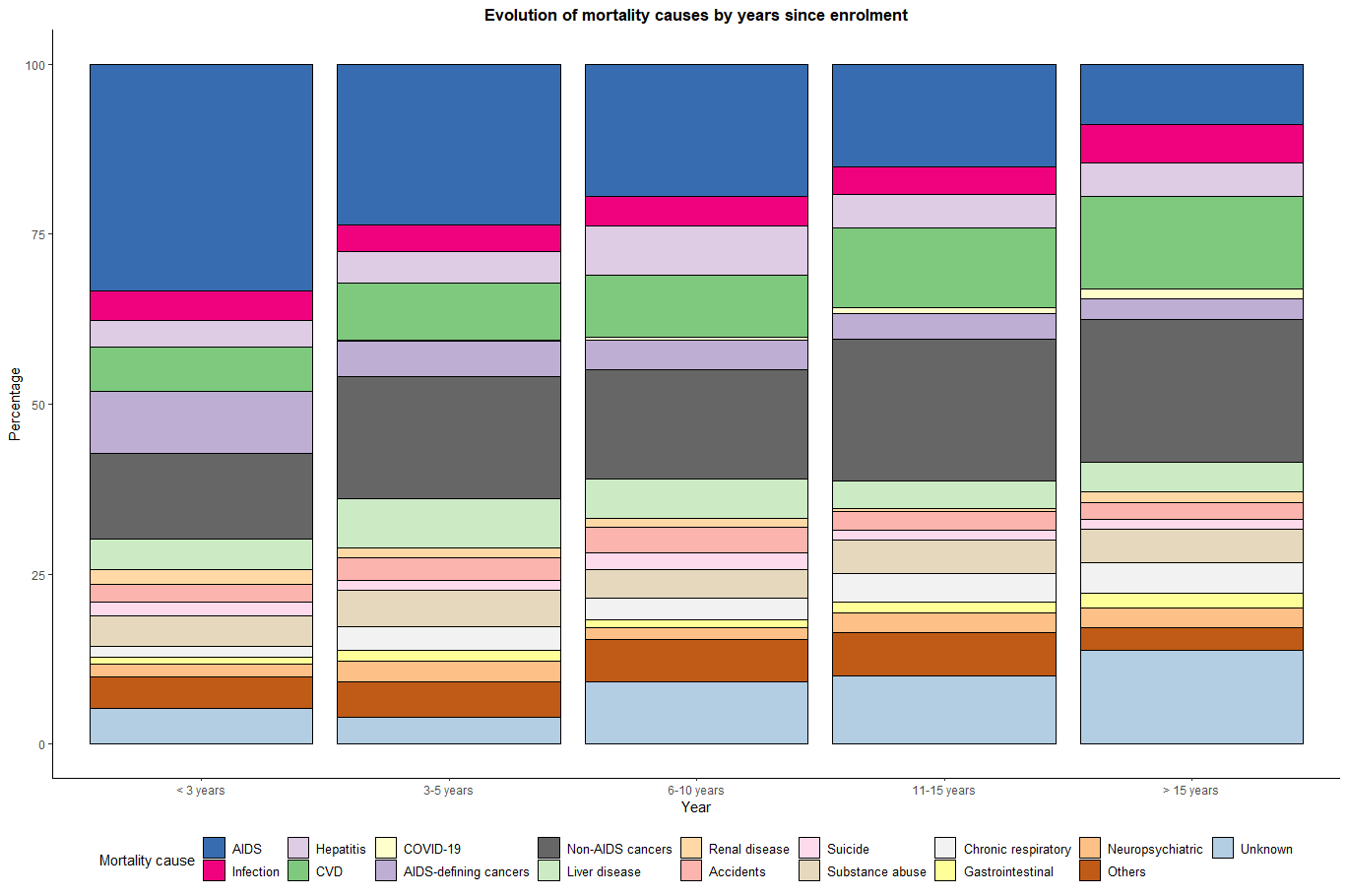


Abbreviations: CVD, cardiovascular disease; COVID-19, coronavirus disease 2019
